# Multimodal Analysis of Composition and Spatial Architecture in Human Squamous Cell Carcinoma

**DOI:** 10.1016/j.cell.2020.05.039

**Published:** 2020-07-23

**Authors:** Andrew L. Ji, Adam J. Rubin, Kim Thrane, Sizun Jiang, David L. Reynolds, Robin M. Meyers, Margaret G. Guo, Benson M. George, Annelie Mollbrink, Joseph Bergenstråhle, Ludvig Larsson, Yunhao Bai, Bokai Zhu, Aparna Bhaduri, Jordan M. Meyers, Xavier Rovira-Clavé, S. Tyler Hollmig, Sumaira Z. Aasi, Garry P. Nolan, Joakim Lundeberg, Paul A. Khavari

**Affiliations:** 1 Program in Epithelial Biology, Stanford University School of Medicine, Stanford, CA 94305, USA; 2 Stanford Cancer Institute, Stanford University School of Medicine, Stanford, CA 94305, USA; 3 Science for Life Laboratory, KTH Royal Institute of Technology, Department of Gene Technology, Tomtebodavägen 23, 171 65 Solna, Sweden; 4 Department of Microbiology & Immunology, Stanford University School of Medicine, Stanford, CA 94305, USA; 5 Institute for Stem Cell Biology and Regenerative Medicine, Stanford University School of Medicine, Stanford, CA 94305, USA; 6 Department of Neurology, University of California, San Francisco (UCSF), San Francisco, CA 94122, USA; 7 Veterans Affairs Palo Alto Healthcare System, Palo Alto, CA, USA

**Keywords:** squamous cell carcinoma, intra-tumoral heterogeneity, scRNA-seq, spatial transcriptomics, MIBI, tumor microenvironment, tumor immunology, skin cancer, multi-omics, CRISPR screen

## Abstract

To define the cellular composition and architecture of cutaneous squamous cell carcinoma (cSCC), we combined single-cell RNA sequencing with spatial transcriptomics and multiplexed ion beam imaging from a series of human cSCCs and matched normal skin. cSCC exhibited four tumor subpopulations, three recapitulating normal epidermal states, and a tumor-specific keratinocyte (TSK) population unique to cancer, which localized to a fibrovascular niche. Integration of single-cell and spatial data mapped ligand-receptor networks to specific cell types, revealing TSK cells as a hub for intercellular communication. Multiple features of potential immunosuppression were observed, including T regulatory cell (Treg) co-localization with CD8 T cells in compartmentalized tumor stroma. Finally, single-cell characterization of human tumor xenografts and *in vivo* CRISPR screens identified essential roles for specific tumor subpopulation-enriched gene networks in tumorigenesis. These data define cSCC tumor and stromal cell subpopulations, the spatial niches where they interact, and the communicating gene networks that they engage in cancer.

## Highlights

• Profiling of 10 human skin SCCs and matched normals via scRNA-seq, ST, and MIBI
• Tumor-specific keratinocytes (TSKs) reside within a fibrovascular niche at leading edges
• Distinct ligand-receptor and spatial niche associations for tumor and stromal cells.
• Subpopulation essential tumorigenic gene networks defined by *in vivo* CRISPR screening

## Graphical Abstract

**Figure:**
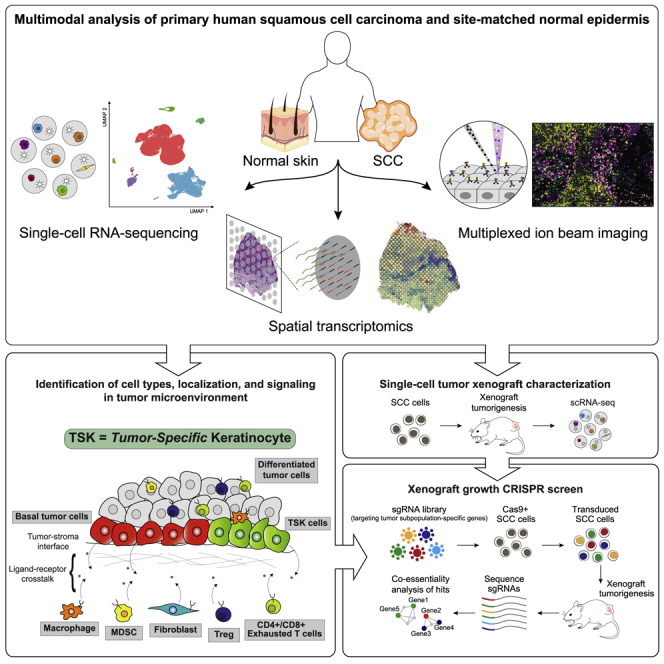

## Teaser

Integration of high-dimensional multi-omics approaches to characterize human cutaneous squamous cell carcinoma identifies a tumor-specific keratinocyte population as well as the immune infiltrates and heterogeneity at tumor leading edges.

## Introduction

Epithelial cancers comprise ~90% of human malignancies. Among these, cutaneous squamous cell carcinoma (cSCC) serves as a prototype, given its cardinal features such as disruption of tissue polarity and basement membrane invasion. cSCC is the second most common malignancy in the U.S., with an annual incidence over 1 million (Rogers et al., 2015). Although excision can be curative, up to 4% of cSCCs develop nodal metastases and approximately 1.5% of patients succumb to the disease (Karia et al., 2013). The recent approval of cemiplimab, an inhibitor of programmed death 1 (PD-1), for advanced and metastatic disease is promising (Migden et al., 2018), but resistance and inadequacy of biomarkers to predict response (Keenan et al., 2019) emphasize the need for better characterization of immunosuppression in the tumor microenvironment (TME).

Single-cell transcriptomics has revealed intra-tumoral heterogeneity (ITH) within many cancer types, identifying cell populations that drive drug resistance, predict metastatic risk, and mediate plasticity (Neftel et al., 2019, Puram et al., 2017, Tirosh et al., 2016a). However, studies often suffer from lack of normal tissue comparisons needed to identify tumor-specific biology and loss of spatial information during tissue dissociation. Recent advances, however, enable simultaneous capture of the locations of dozens of cell types within the TME, which is critical for understanding tumor-stroma crosstalk (Angelo et al., 2014, Keren et al., 2018, Salmén et al., 2018a, Thrane et al., 2018). Orthogonal integration of single-cell and high-dimensional spatial data from both normal and diseased tissue should therefore facilitate the dissection of TME cellular communication.

Here, we combine single-cell and spatial transcriptomics (ST) with single-cell resolution multiplexed protein imaging of a series of primary human cSCCs, along with matched normal skin. 48,164 single-cell transcriptomes were characterized from 10 tumor-normal skin pairs using single-cell RNA sequencing (scRNA-seq). These data were integrated with ST profiling of 17,064 spots and with multiplexed ion beam imaging (MIBI) of 55,832 cells. Tumors included basal, cycling, and differentiating keratinocyte (KC) populations similar to normal skin, along with a tumor-specific keratinocyte (TSK) population. TSK cells localized to leading edges, as did a population of basal tumor cells. TSK, basal, and adjacent stromal and immune cell types exhibited invasive and immunosuppressive features associated with physical proximity and distinct sets of ligands and receptors. Xenograft tumors and TME recapitulated spontaneous human cSCC, facilitating *in* vivo CRISPR screens that identified an essential tumorigenic function for TSK-enriched integrin signaling genes *ITGB1*, *FERMT1*, and *CD151*. Together, these results uncover cSCC spatial heterogeneity, highlight potential intercellular signals controlling the positioning and states of associated cell types, and serve as a resource for further investigation of tumor and immune dynamics.

## Results

### Multimodal Profiling of cSCC

**Figure S1:**
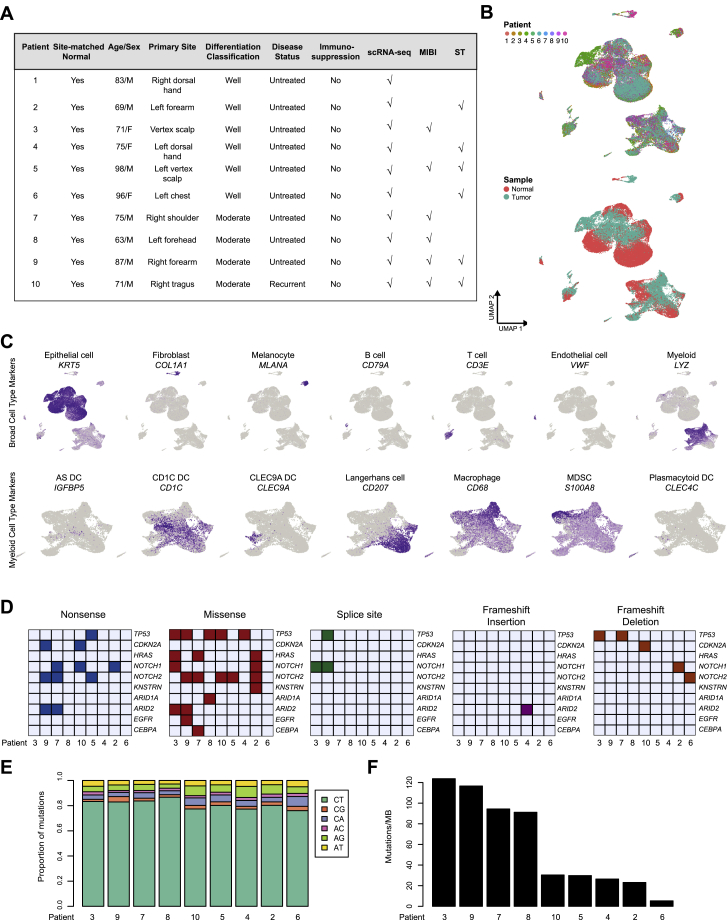
scRNA-seq and Whole-Exome Sequencing of Normal Skin and cSCC, Related to Figure 1 (**A**) Patient cohort characteristic table. (**B**) UMAP of all cells (*k* = 48,164) recovered labeled by patient on the top and tissue type (normal skin or tumor) on the bottom. (**C**) UMAP feature plots showing expression of marker genes by annotated cell types. (**D**) Types of mutations in select genes in patient cSCC samples. (**E**) Proportion of transitions and transversions mutations across patients. (**F**) Tumor mutational burden across patients. MB = megabase.

**Figure 1:**
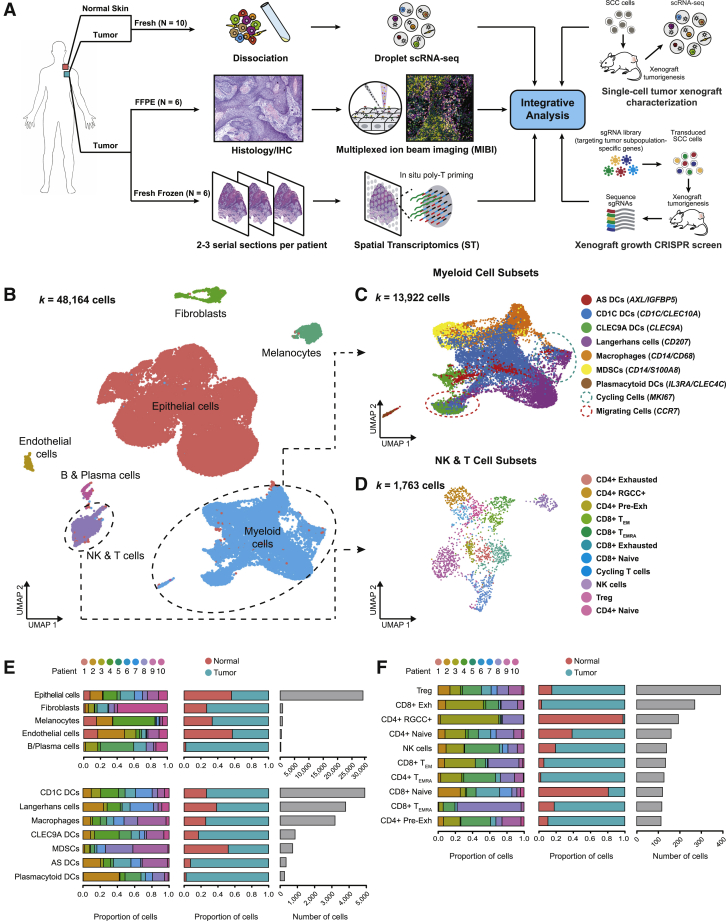
A Single-Cell Transcriptomic Atlas of Normal Skin and cSCC (A) Workflow of patient sample processing for scRNA-seq, MIBI, and ST with integration of a xenograft CRISPR screen. (B) Uniform manifold approximation and projection (UMAP) of scRNA-seq cells recovered from both normal skin and cSCC labeled by cell type. (C) UMAP of myeloid subsets, including dendritic cells (DCs), macrophages, and monocytic myeloid-derived suppressor cells (MDSCs) (marker genes in parentheses). (D) UMAP of subsets of natural killer (NK) and T cell subsets, including CD4^+^ and CD8^+^ conventional T cells and associated subsets, and regulatory T cells (Treg). Pre-Exh, pre-exhausted; T_EM_, effector memory T cell; T_EMRA_, recently activated effector memory T cell. (E) Bar plots of proportion of cell type by patient, tumor or normal origin, and total cell number. (F) Similar to (E), for NK cell and T cell subsets. See also Figure S1 and Table S1.

We performed scRNA-seq on tumors and patient- and site-matched normal skin collected from 10 individuals presenting for surgical resection using the 10X Genomics Chromium platform (Figure S1A; STAR Methods). Prior to dissociation, portions of tumors were fresh frozen and formalin-fixed for ST (Salmén et al., 2018b, Ståhl et al., 2016) and MIBI (Angelo et al., 2014), respectively (Figure 1A). 50,009 cells were sequenced at a median depth of 115,216 reads/cell, with 48,164 cells passing filtering utilizing Seurat (Butler et al., 2018) (Table S1; STAR Methods). Unsupervised clustering identified tumor and normal epithelial cells as well as stromal subtypes (Figure 1B; STAR Methods). In addition to fibroblasts, endothelial cells, and melanocytes, the latter included CLEC9A, CD1C, plasmacytoid, and AXL^+^SIGLEC6^+^ (AS) dendritic cells (DCs) (Villani et al., 2017), Langerhans cells, macrophages, and myeloid-derived suppressor cells (MDSCs) (Figures 1C and S1C). Natural killer (NK) and T cell clusters included CD4 and CD8 conventional and exhausted T cells, regulatory T cells (Treg), and naive-like T cells (Figure 1D), all bearing resemblance to subsets from prior solid tumor scRNA-seq studies (Guo et al., 2018, Puram et al., 2017, Savas et al., 2018, Tirosh et al., 2016a, Yost et al., 2019, Zhang et al., 2018, Zheng et al., 2017). Non-tumor clustering was driven predominantly by cell type rather than batch effects (Figures S1B and S1C). Cell-type proportion analysis reflected consistent sample processing (Figures 1E and 1F). Whole-exome sequencing found a mutational landscape consistent with prior cSCC studies (Lee et al., 2014, Li et al., 2015, Pickering et al., 2014, South et al., 2014) (Figures S1D–S1F). Taken together, these data provide a representative cellular atlas for cSCC.

### A TSK Subpopulation

**Figure 2:**
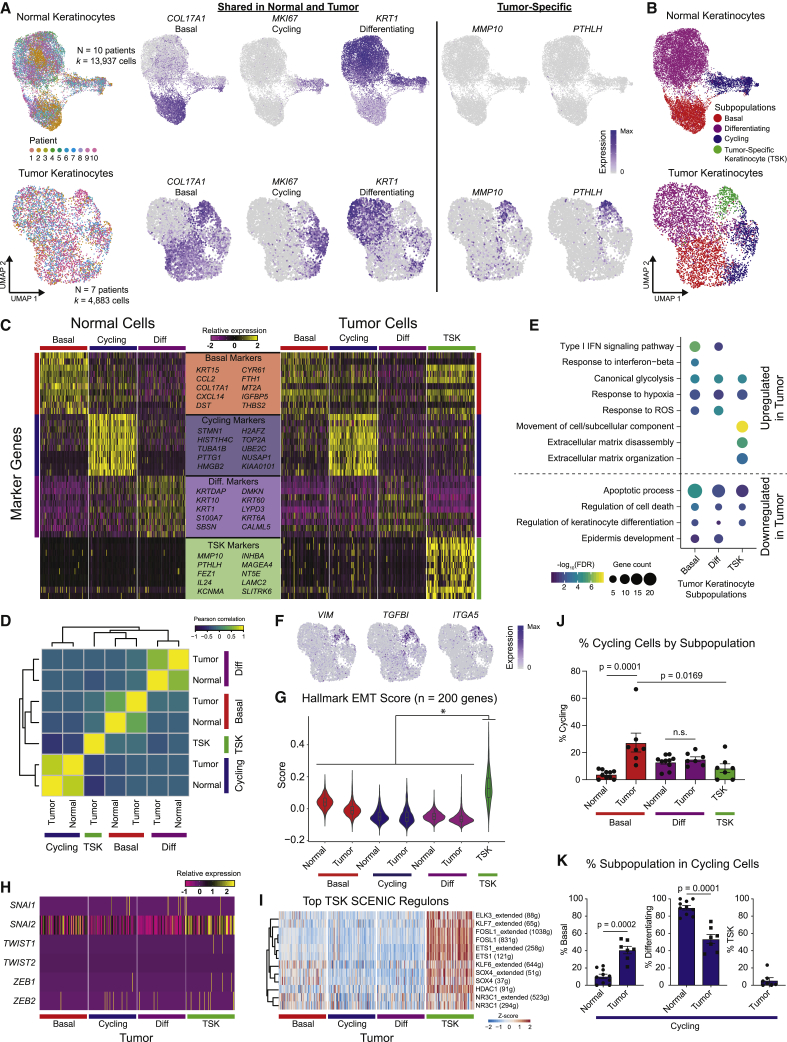
A Dysregulated Differentiation Hierarchy in Tumor Keratinocytes (A) Left, UMAP of normal keratinocytes (KCs) of the interfollicular epidermis and tumor KCs labeled by patient; middle, expression of basal, cycling, and differentiating genes found in both normal and tumor KCs; right, expression of tumor-specific genes. (B) UMAP classifying normal and tumor KCs into analogous basal, cycling, and differentiating clusters. Tumors contain a tumor-specific keratinocyte (TSK) subpopulation. (C) Expression of top 10 shared basal, cycling, differentiating, and TSK marker genes. TSK genes are minimally expressed in normal KCs. (D) Correlation matrix of overlapping differentially expressed genes (STAR Methods). (E) Dot plot of gene ontology (GO) terms for top 200 up- and downregulated genes in subpopulation differential expression for tumor versus normal KCs. TSK was compared to normal basal. (F) UMAP feature plots of expression for genes in hallmark epithelial-mesenchymal transition (EMT) signature in tumor KCs. (G) Violin plots of the hallmark EMT gene signature score in normal and tumor KC subpopulations. ^∗^p < 2.2 × 10^−16^ by pairwise Wilcoxon rank-sum tests with Benjamini-Hochberg correction. (H) Heatmap of expression of EMT-associated transcription factors in tumor KCs. (I) Heatmap of single-cell regulon scores inferred by SCENIC (g, genes; extended, SCENIC-annotated additional genes). (J) Bar plots of percentage of cycling cells per KC subpopulation after regressing cell-cycle signature from cycling cells. (K) Bar plots of percentage of subpopulation representation of KC cycling cells after cell-cycle regression. Data shown in (J) and (K) are averages ±SEM. Normal, n = 10 patients; tumor, n = 7 patients. p values were determined by Mann-Whitney U test. For visualization, a random sampling of 100 cells per subpopulation are shown in (C), (H), and (I). See also Figure S2 and Tables S2 and S3.

**Figure S2:**
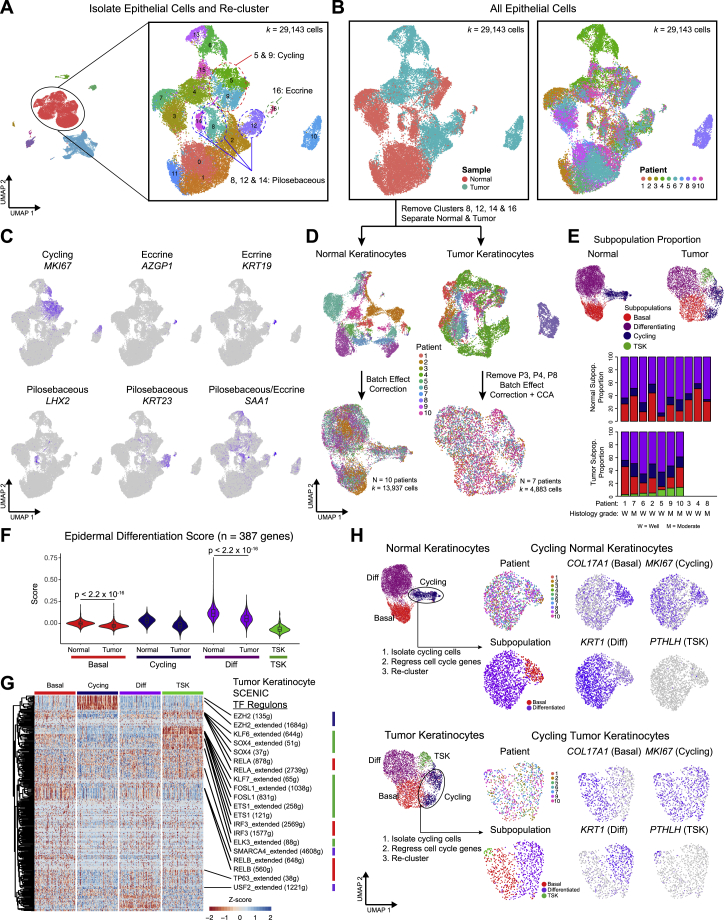
Keratinocyte Annotation and Subpopulation Analysis, Related to Figure 2 (**A**) UMAP of all epithelial cell clusters (*k* = 29,143) with labeled cycling, eccrine cell, and pilosebaceous clusters. (**B**) UMAP of all epithelial cells labeled by tissue type and patient. (**C**) UMAP feature plots of expression of genes marking cycling, eccrine, or pilosebaceous clusters. (**D**) UMAPs of filtered normal and tumor keratinocytes labeled by patient before and after batch correction. CCA = canonical correlation analysis. (**E**) Bar plots showing proportion of each subpopulation across normal and tumor keratinocytes. (**F**) Violin plots of differentiation signature score (n = 387 genes, Lopez-Pajares et al., 2015) in normal and tumor keratinocyte subpopulations. p values were determined with pairwise Wilcoxon Rank Sum tests with Benjamini-Hochberg correction for multiple comparisons. (**G**) Clustered heatmap of all recovered transcription factor (TF) regulons from SCENIC analysis (n = 370 regulons) across tumor keratinocyte subpopulations. For visualization purposes, 100 random cells from each subpopulation are shown in the heatmap. (**H**) Cell cycle regression analysis with re-clustering into basal, differentiating, and TSK subpopulations with UMAP feature plots showing marker gene expression after cell cycle regression.

After re-clustering normal epithelial cells, three major KC subpopulations were observed in normal skin, each expressing known representative genes, including (1) basal (*COL17A1*), (2) cycling (*MKI67*), and (3) differentiating (*KRT1*) cells (Figures 2A and S2A–S2D; STAR Methods). These correspond to known functions of cells within the epidermis, and their presence in cSCCs was next examined. Canonical correlation analysis (Butler et al., 2018) demonstrated that tumor KCs broadly recapitulated the normal (basal, cycling, and differentiating) subpopulations, with several shared marker genes across normal skin and cSCC (Figures 2A–2D and S2D; Table S2). Interestingly, clustering of tumor cells yielded a fourth major KC subpopulation with no clear correlate in normal skin (Figures 2B–2D). Indeed, out of the 100 genes distinguishing this cluster from other tumor cells, 99 were expressed significantly higher in tumors compared to normal tissue (log fold change >0.25 and p < 5.4 × 10^−24^; Table S3). This TSK subpopulation comprised 2.7% to 13.8% of tumor cells across patients (Figures S2D and S2E). Although exhibiting shared marker genes, tumor KC subpopulations differed from their normal counterparts in some ways, including upregulation of type I interferon response, glycolysis, and hypoxia genes in basal tumor KCs and downregulation of apoptotic and terminal differentiation genes in the differentiating tumor cells (Figures 2E and S2F). Thus, cSCCs contain KCs that generally recapitulate the major cell states in normal epidermis as well as a tumor-specific TSK subpopulation with no counterpart in normal skin.

TSK signature genes were linked to cellular movement and extracellular matrix disassembly, suggesting invasive behavior (Figure 2E). Consistent with this, TSKs expressed classic epithelial-mesenchymal transition (EMT) markers such as *VIM* and *ITGA5* (Figure 2F). Furthermore, TSKs exhibited the highest expression of the Hallmark EMT gene signature (n = 200 genes, p < 2.2 × 10^−16^) (Figure 2G; STAR Methods) (Liberzon et al., 2015). Similar to a previous study of oropharyngeal SCC (Puram et al., 2017), EMT-like TSK cells lacked expression of classic EMT transcription factors (TFs) (Figure 2H). Therefore, we performed single-cell regulatory network inference and clustering (SCENIC) (Aibar et al., 2017), which nominated AP1 and ETS family members as TFs potentially controlling TSKs (Figures 2I and S2G). TSK cells also exhibited a broad range of EMT scores, suggesting high cell state plasticity (Figure 2G), consistent with the model of an EMT continuum (Lambert et al., 2017, McFaline-Figueroa et al., 2019, Nieto et al., 2016, Pastushenko et al., 2018, Puram et al., 2017).

Finally, we found that basal tumor cells proliferated roughly five times more frequently than basal cells in normal tissue (p = 1 × 10^−4^) (Figure S2H; STAR Methods). Conversely, tumor and normal differentiating KCs exhibited no differences in cycling (Figure 2J), possibly reflecting a requirement for cell-cycle exit in terminal differentiation (Jones et al., 2007). TSK cells cycled the least frequently in tumors (∼8%), and basal cells were approximately four times more common in tumor than normal cycling cells (p = 2 × 10^−4^) (Figure 2K). In sum, these data point to an epidermal differentiation hierarchy in cSCC that is dysregulated in key aspects: (1) failure to fully engage differentiation, (2) rapidly proliferating basal cells, and (3) the emergence of a TSK subpopulation expressing EMT-linked genes.

### Spatial Transcriptomics Identifies TSK-Basal Heterogeneity at the Leading Edge

**Figure S3:**
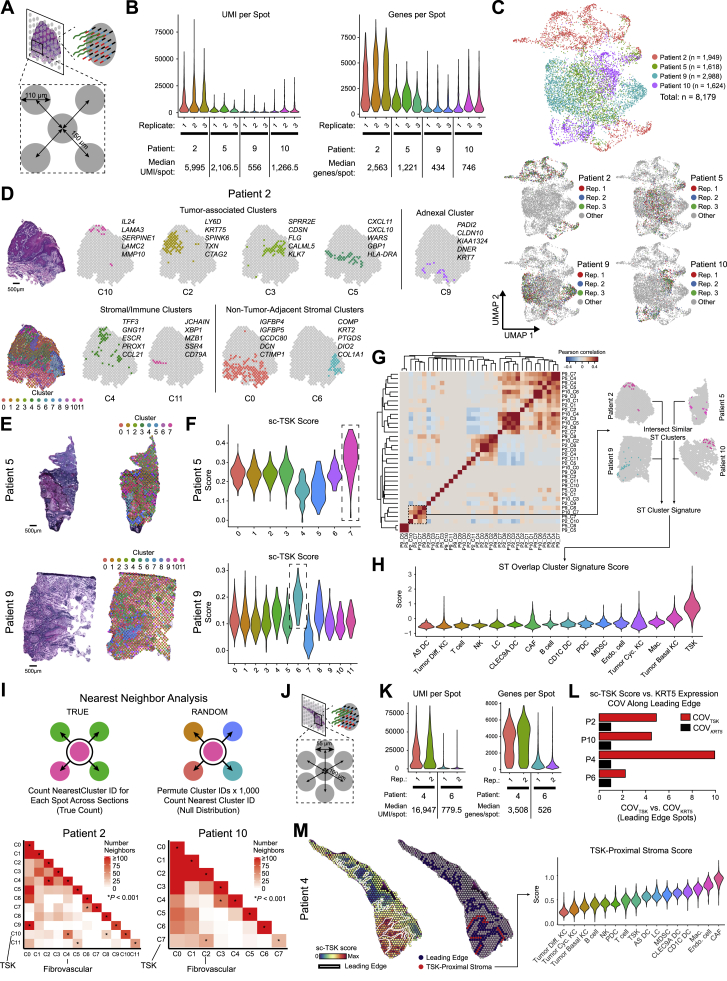
Spatial Transcriptomics Identifies TSK Localization and Patterns of Cluster Adjacency, Related to Figure 3 (**A**) Spatial transcriptomics (ST) spot size and resolution. (**B**) Violin plots of UMI counts per spot and genes per spot across tissue section replicates. (**C**) UMAP of all transcriptome spots labeled by patient (top) and replicate (bottom). (**D**) Tumor-associated spot clusters (clusters encompassing annotated tumor regions in sections), stromal or immune-associated, and non-tumor-adjacent stromal and adnexal spot clusters projected individually with labeled top differentially expressed genes. (**E**) Hematoxylin and eosin (H&E) staining of sections from Patients 5 and 9 with unbiased clustering of spots based on global gene expression within individual spots. Scale bar = 500 μm (**F**) Violin plots of TSK scores of individual spots derived from scRNA-seq data (sc-TSK score) for each cluster. Dotted boxes outline clusters with highest average sc-TSK score. (**G**) and (**H**) Overlap correlation matrix of genes differentially expressed in ST clusters across all patients (G). Highlighted similar spatial clusters were used to generate ST Cluster Signature (n = 6 genes), and violin plots of ST Cluster Signature score by cell types in scRNA-seq data (H). (**I**) Top, schematic of nearest neighbor analysis for spots. Bottom, heatmaps showing number of nearest neighbor identities for each cluster. ^∗^indicates p < 0.001 by permutation test. (**J**) Visium platform ST spot size and resolution. (**K**) Violin plots of UMI counts per spot and genes per spot across tissue section replicates from Visium. (**L**) Coefficient of variation of sc-TSK score (COV_TSK_) normalized to COV of KRT5 expression (COV_*KRT5*_) across leading edge spots in patient ST data. (**M**) Left, spatial feature plot of sc-TSK score. Middle, spots annotated by leading edge (navy) and TSK-proximal stroma (red). Differential expression was performed with TSK-proximal stroma spots versus all other spots in tissue. Right, Violin plots of TSK-proximal stroma scores (n = 209 genes, Wilcoxon rank-sum test, adjusted p value < 0.05) by cell types in scRNA-seq data (STAR Methods).

**Figure 3:**
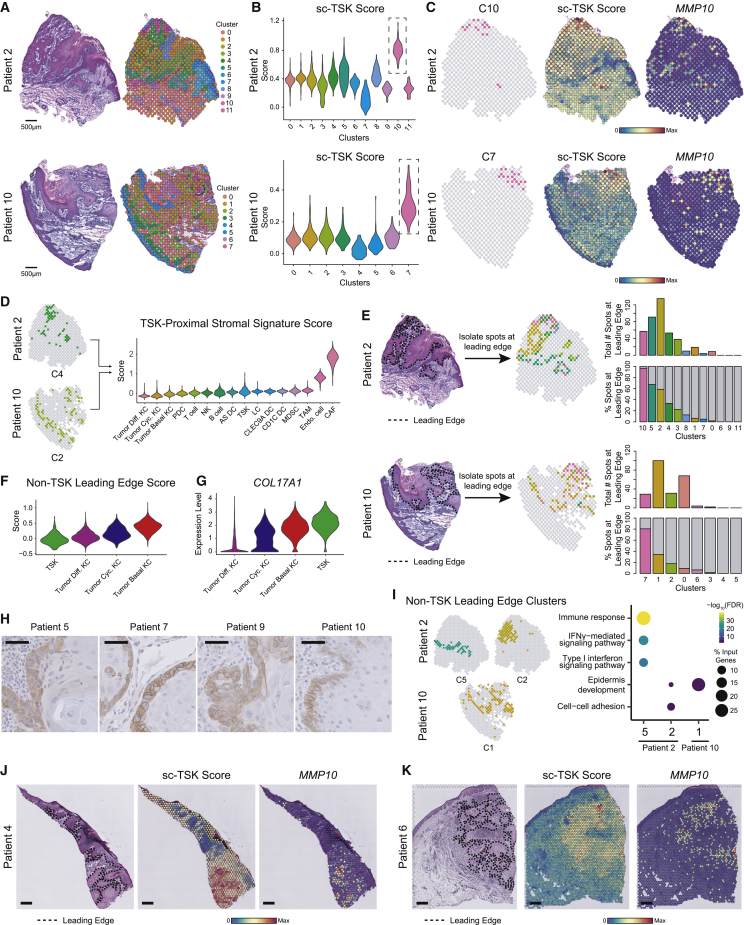
Leading Edge Heterogeneity Revealed by Spatial Transcriptomics (A) Hematoxylin and eosin (H&E) staining of tissue sections and unbiased clustering of ST spots. Scale bar, 500 μm. (B) Violin plots of TSK scores of individual spots derived from scRNA-seq data (sc-TSK score) for each cluster. Dotted boxes outline clusters with highest average sc-TSK score. (C) Spatial feature plots of TSK-high cluster, sc-TSK score, and TSK marker *MMP10* expression in tissue sections. (D) Violin plots of TSK-proximal signature score (intersection of differentially expressed genes in patient 2 cluster 4 and patient 10 cluster 2, n = 34 genes) for cell types in scRNA-seq data. (E) Left, H&E staining with leading edge of tumor annotated (dotted lines) and isolated leading edge spots labeled by cluster. Right, bar plots of total number and percentage of spots at leading edge per cluster. (F) Violin plots of non-TSK leading edge signature score (intersection of differentially expressed genes of non-TSK spots at leading edge from patient 2 and patient 10, n = 6 genes) by tumor subpopulations. (G) Violin plots of *COL17A1* expression by tumor subpopulation in scRNA-seq data. (H) Immunohistochemical staining of COL17A1 in patient tumors. Scale bar, 50 μm. (I) Projection of non-TSK leading edge-associated clusters with dot plot of select gene ontology (GO) terms of differentially expressed genes in each cluster (n = 200 genes for patient 2 clusters, n = 20 for patient 10 cluster). (J and K) H&E, sc-TSK score, and *MMP10* expression feature plots from (J) patient 4 and (K) patient 6 with data generated using the Visium ST platform. Scale bar, 500 μm. KC, keratinocyte; TAM, tumor-associated macrophage. See also Figure S3 and Table S4.

To assess the spatial organization of tumor cell populations, we performed ST on triplicate sections from a subset of tumors (Figure S3A). Transcriptomes from 8,179 spots across 12 sections were obtained at a median depth of 1,629 UMIs/spot and 967 genes/spot (Figures S3B and S3C). Across patients, tumor-associated spot clusters exhibited expression of genes mapping to tumor KCs in scRNA-seq, while immune or stromal genes were associated with tumor-adjacent stroma, uninvolved stromal, or adnexal areas, consistent with gross histologic cSCC architecture (Figures 3A, S3D, and S3E; Table S4).

Scoring spots in each section with TSK-signature genes from scRNA-seq (sc-TSK score, Table S3; STAR Methods) highlighted a single clear TSK-high cluster, including the TSK marker *MMP10*, in each patient (Figures 3B, 3C, and S3D–S3F). These TSK-high clusters were similar across patients (Figures S3G and S3H; Table S4). In tumors with clear leading edges, the TSK-adjacent stroma (C4 in patient 2, C2 in patient 10), was enriched for cancer-associated fibroblast (CAF) and endothelial transcripts, highlighting a fibrovascular niche (Figures 3D and S3I).

Over 80% of TSK-high cluster spots were located at tumor leading edges, although they comprised a minority of total spots in this compartment; the remaining leading edge spots were enriched for basal tumor genes (Figures 3E and 3F). The presence of both tumor basal and TSK cells along the leading edge was further supported by immunohistochemical (IHC) staining for COL17A1, a shared tumor basal and TSK marker (Figures 3G and 3H). Additionally, inflammatory response and interferon signaling genes were expressed in the non-TSK leading edge, consistent with the presence of interferon associated transcripts in the tumor basal population (Figures 2E and 3I).

Finally, we generated an additional 8,885 spot transcriptomes utilizing the Visium Spatial Gene Expression kit (10X Genomics) at higher spot resolution and density from two additional patient tumors (Figures S3J and S3K). In these samples, we also observed high sc-TSK scores and heterogeneity along the leading edge (Figures 3J, 3K, and S3L). Analysis of stroma at a distance of one spot (∼100 μm) away from the TSK-high leading edge demonstrated an enrichment of endothelial and CAF-associated transcripts, further supporting a fibrovascular niche surrounding TSK cells (Figure S3M). Collectively, these data identify a heterogeneous cSCC tumor leading edge composed of TSK and basal tumor cells and a TSK-proximal fibrovascular niche.

### The Immune Landscape of cSCC

**Figure 4:**
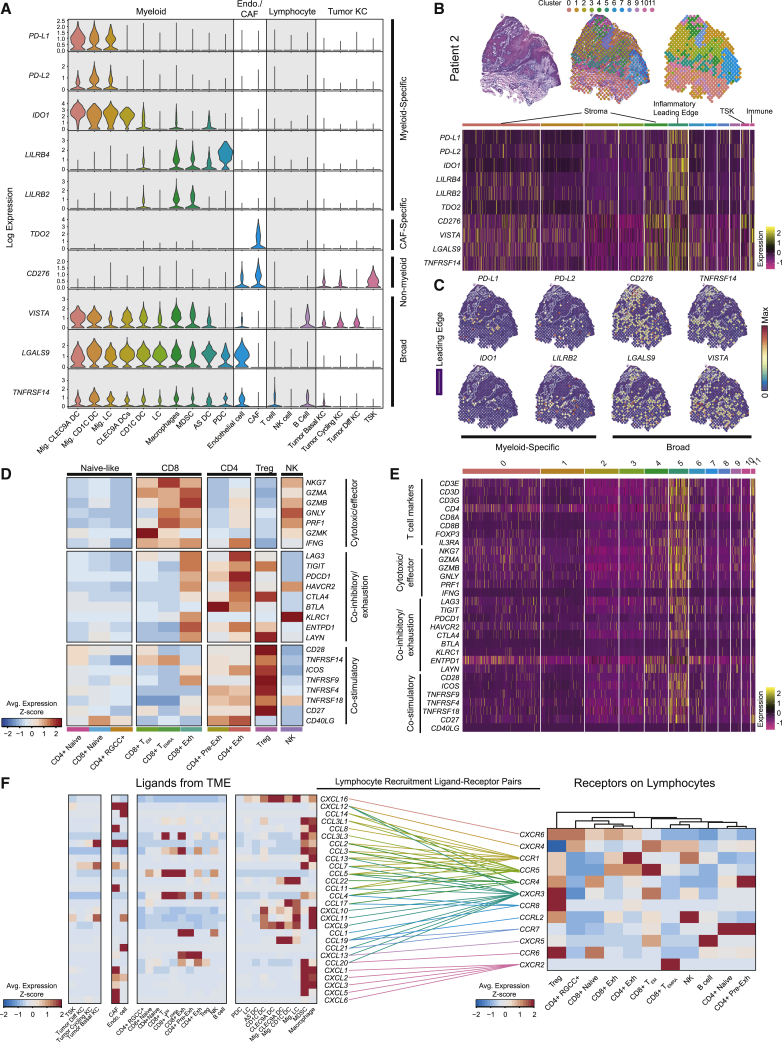
The Immune Landscape of cSCC (A) Violin plots of select immunosuppression-associated gene expression across cell types of the TME from scRNA-seq data. (B) Top, spatial transcriptomics (ST) cluster map from patient 2. Bottom, heatmap of select immunosuppression-associated gene expression across spatial transcriptomic spots grouped by cluster. (C) Feature plots of select immunosuppression-associated genes by ST. (D) *Z*-scored mean log expression heatmap of genes associated with cytotoxicity, exhaustion, and co-stimulatory function across T cell subsets in scRNA-seq data. (E) Heatmap of expression of T cell marker genes, and genes associated with cytotoxicity, exhaustion, and co-stimulatory function in ST spots grouped by cluster. (F) Left, *Z*-scored mean log expression heatmap of chemokine genes across cell types from scRNA-seq data. Right, *Z*-scored mean log expression heatmap of chemokine receptor genes across lymphocyte cell types from scRNA-seq data. Colored lines connect matching ligands for each chemokine receptor. Pre-Exh, pre-exhausted; T_EM_, T effector memory; T_EMRA_, T recently activated effector memory; Mig., migrating; DC, dendritic cell; CAF, cancer-associated fibroblast; KC, keratinocyte. See also Figure S4.

To explore how distinct cell types may contribute to immune activity in cSCC, the expression of known immunosuppressive genes was surveyed (Figure 4A). *CD274* (*PD-L1*), and *PDCD1LG2* (*PD-L2*) were exclusively expressed in migrating DCs, suggesting DCs in the draining lymph node may limit anti-PD-1 therapy response, possibly explaining the observation of novel T cell clone infiltration into tumors after anti-PD-1 therapy (Ho et al., 2019, Yost et al., 2019). Similar DCs recently described in a murine model and patients with lung adenocarcinoma were coined “mature DCs enriched in immunoregulatory molecules” (mregDCs) (Maier et al., 2020). Migrating DCs also upregulated *IDO1*, which inhibits T cell cytotoxic activity and promotes differentiation into Tregs (Munn and Mellor, 2016). Interestingly, *CD276* (B7-H3), known to augment Tregs and inhibit T cell responses (Costa et al., 2018, Prasad et al., 2004, Suh et al., 2003), was expressed most prominently on CAFs and TSKs. Several genes, including *C10orf54* (*VISTA*), *LGALS9*, and *TNFRSF14*, known to inhibit T cells (Benci et al., 2019, Chen and Flies, 2013) were broadly expressed. These genes exhibited spatial cluster-specific patterns in ST, consistent with the co-localization of particular cell types, with myeloid-specific factors predominant at an inflammatory leading edge (Figures 4B and 4C).

**Figure S4:**
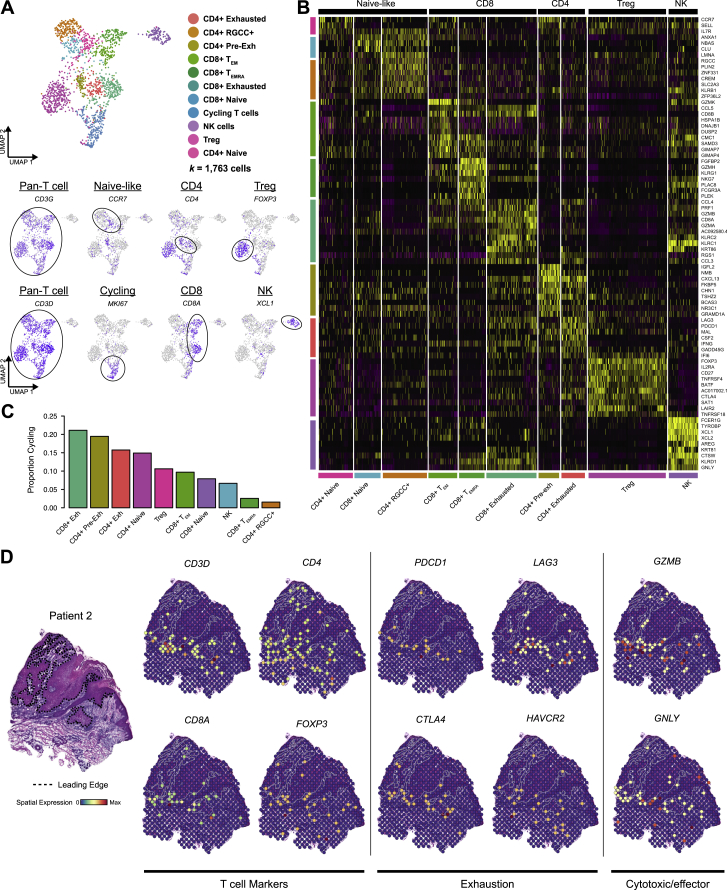
T Cell Subset Characterization and Spatial Positioning, Related to Figure 4 (**A**) UMAP of T cell subsets and NK cells with feature plots showing expression of subset marker genes. (**B**) Heatmap of top differentially expressed genes by NK cells and T cell subsets in scRNA-seq data. (**C**) Bar plots of proportion of cycling cells by NK or T cell subset. (**D**) Spatial feature plots showing expression of select genes grouped by function from Patient 2.

Exhausted CD4 and CD8 T cell subsets expressed inhibitory receptors and exhaustion markers, such as PD-1 (*PDCD1)*, *CTLA4*, *LAG3*, *TIGIT*, and *HAVCR2*, in addition to cytotoxic genes such as *GZMA/B*, *GNLY*, and *PRF1* (Figures 4D, S4A, and S4B). Similar effector and exhausted T cells, which have the highest proliferative capacity among T cell subsets, were observed in other cancers (Figure S4C) (Guo et al., 2018, Li et al., 2019, Savas et al., 2018, Thommen et al., 2018, Tirosh et al., 2016a, Zheng et al., 2017). CD4 exhausted T cells expressed follicular helper-associated molecules *ICOS* and *CXCL13* (Figures 4D and S4B), resembling cells shown to mediate responses to CTLA-4 blockade (Wei et al., 2017). Spatially, T cell transcripts appeared in the inflammatory leading edge and immune-associated clusters (Figures 4E and S4D).

Potential recruitment mechanisms for tumor-infiltrating lymphocytes were next assessed by analyzing chemokine and receptor expression in scRNA-seq (Figure 4F). Several chemokine receptors were highly expressed across overlapping T cell subsets, such as *CXCR3* (Treg, CD8^+^ T_EM_), *CXCR6* (Treg, CD4^+^ RGCC^+^, and CD8^+^ exhausted), and *CXCR4* (CD4^+^ RGCC^+^, CD8^+^ T_EM_, CD8^+^ T_EMRA_, and NK cells), suggesting co-recruitment and consistent with co-localization in ST (Figures 4E and S4D). Tregs specifically expressed *CCR8*, nominating it as potential therapeutic target for inhibiting Treg recruitment. Macrophages and/or MDSCs highly expressed several potential mediators of Treg recruitment, including *CXCL9*/*10*/*11* (to *CXCR3*), *CCL4* (to *CCR4*/*8*), and *CCL20* (to *CXCR3* and *CCR6*), consistent with prior studies (Campbell and Koch, 2011, Liu et al., 2011, Redjimi et al., 2012). Taken together, these results highlight multiple cell types involved in immunosuppressive mechanisms that include induction of co-inhibitory signals on DCs and exhausted T cells and recruitment of Tregs.

### Single-Cell Spatial Architecture of the Inflammatory TME

**Figure 5:**
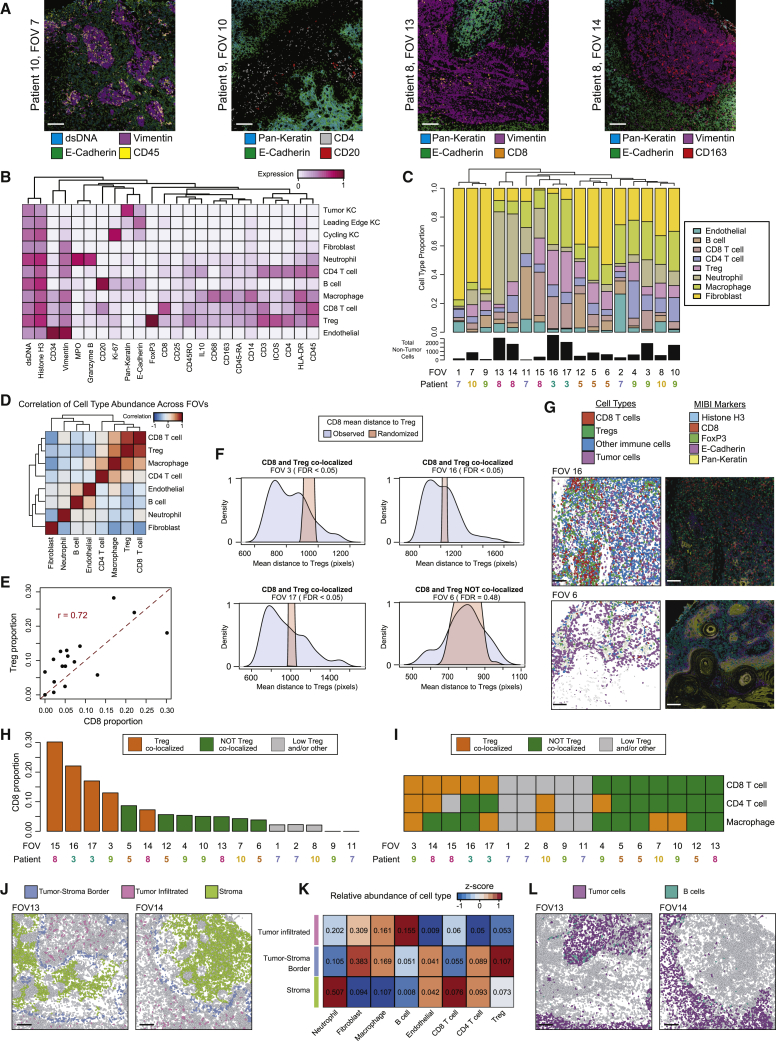
Spatial Architecture of Lymphocyte Subsets in cSCC (A) Select MIBI fields of view (FOVs) for patient samples with expression of highlighted features. (B) Heatmap of feature expression across cell types identified by MIBI. (C) Top, bar plots of proportion of non-tumor cell types across all FOVs. Bottom, bar plots of total numbers of non-tumor cells identified in each FOV. (D) Correlation heatmap of non-tumor cell types across all FOVs. (E) Scatterplot and correlation of CD8 T cell and regulatory T cell (Treg) correlation across FOVs. (F) Density plots of CD8 T cell distance to Tregs across four FOVs, demonstrating variation in CD8 to Treg co-localization. FDR, false discovery rate by permutation (STAR Methods). (G) Expression of Treg marker FoxP3, CD8, and tumor cell markers E-cadherin and pan-keratin. (H) Ranked bar plots of CD8 proportion in each FOV labeled by co-localization pattern with Tregs. (I) Co-localization patterns of Tregs and either CD4, CD8, or macrophages. (J) Non-tumor cells flagged by location relative to tumor and stromal compartments. (K) Heatmap of relative abundance of cell types in each compartment. Values represent proportion of total non-tumor cells in compartment contributed by each cell type. (L) B cells infiltrate tumor parenchyma in select FOVs. All FOVs in figure are 800 × 800 μm. All scale bars, 100 μm. See also Figure S5 and Table S5.

**Figure S5:**
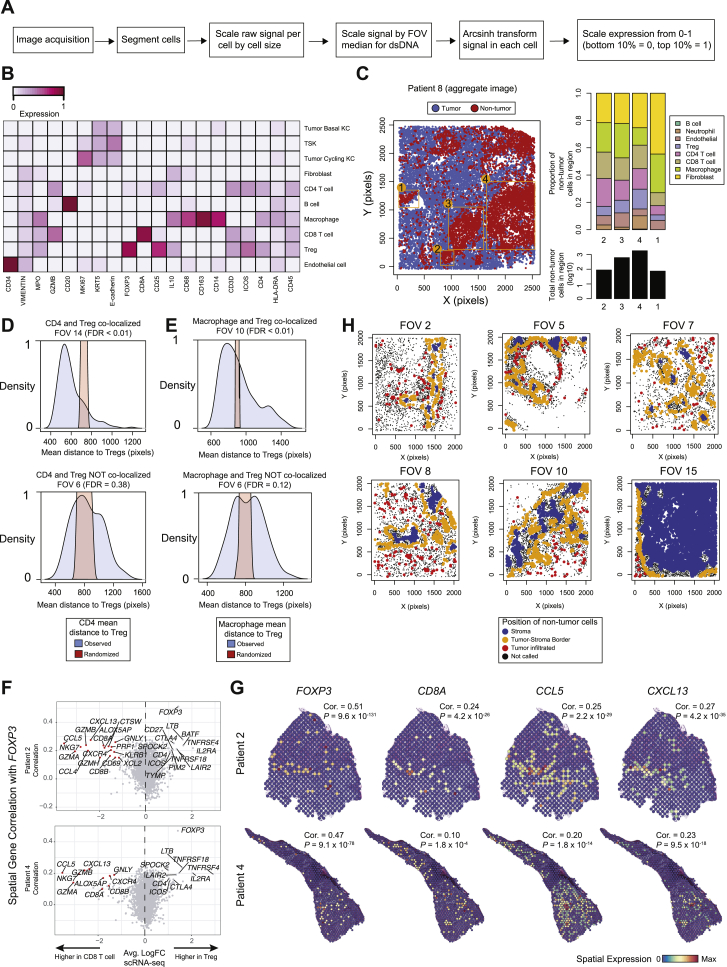
Multiplexed Ion Beam Imaging Acquisition and Analysis, Related to Figure 5 (**A**) Workflow for image acquisition and standard processing to achieve marker quantification for single cells. (**B**) Heatmap representing scRNA-seq expression for genes exhibiting similar expression patterns in MIBI and scRNA-seq. (**C**) Plot of cell positions and tumor or normal status in large, tiled image from Patient 8 (2mm x 2mm). Regions outlined by squares are analyzed for composition of non-tumor cells (similar to Figure 5C). (**D**) Distribution of mean distance to Tregs for each CD4 T cell in blue. In red, CD4 cell identities were permuted among all non-tumor, non-CD4, non-Treg cells and the mean distance to Treg was re-calculated. (**E**) Similar to (D), analyzing macrophage to Treg distances. (**F**) Comparison of CD8 versus Treg differential expression and correlation with FOXP3 across ST spots for patients 2 and 4. (**G**) Spatial feature plots of Treg marker FOXP3 and CD8 effector/chemokine genes demonstrating co-localization. (**H**) Similar to Figure 5K. Cells were labeled as stromal (tumor-excluded), leading edge (tumor-stroma border), and infiltrated in tumor parenchyma.

To dissect the spatial organization of the TME at single-cell resolution, MIBI was performed across 18 fields of view (FOVs) encompassing tumor leading edges in six patients. Images of 38 tumor and stromal/immune protein markers were segmented to identify 55,832 cells across all FOVs (Figures 5A and S5A; Table S5; STAR Methods). Clustering classified major cell types that were similar to scRNA-seq cell types (Figures 5B and S5B; STAR Methods). Stromal and immune composition was variable within and across tumors, as 12/17 FOVs did not cluster consistently by patient (Figure 5C). Similarly, aggregated adjacent FOVs in a single section identified four distinct stromal regions across which fibroblasts ranged from 21.6% to 44.6%, neutrophils varied from 0% to 9.7%, and CD4 T cells ranged from 6.8% to 20.5% (Figure S5C).

Despite considerable regional heterogeneity, deeper analysis revealed a high correlation of CD8 T cells, Tregs, macrophages, and CD4 T cells; CD8 T cells and Tregs were especially linked (r ∼0.72) (Figure 5D). Treg and effector cell proximity in secondary lymphoid organs influences immunosuppressive function (Liu et al., 2015), and, similarly, we observed co-localization between Tregs and CD8 T cells in 5/12 FOVs with sufficient numbers of cells for systematic analysis of distance between Tregs and CD8 T cells (Figures 5F–5H; STAR Methods). The proportion of CD8 T cells was also positively associated with co-localization, suggesting Tregs may be regulated both by co-recruitment to the TME and co-localization in CD8 T cell niches. This model is consistent with shared recruitment chemokines between CD8 T cells and Tregs (Figure 4F) as well as CD8 T cell requirement for Treg-mediated suppression (Spranger et al., 2013). While CD4 T cells and macrophages were also correlated in abundance with Tregs, they exhibited co-localization with Tregs in distinct FOVs compared to CD8 T cells (Figures 5I, S5D, and S5E).

In line with MIBI findings, we also observed significant spatial correlation between CD8 T cell markers (*CD8A*/*B*) and the Treg marker *FOXP3* in ST (Figure S5F; STAR Methods). Furthermore, *FOXP3* was spatially correlated with a set of effector molecules (*CCL4*, *CCL5*, *GZMA/B*, *GNLY*), suggesting Tregs home to CD8 T cells with effector activity (Figures S5F and S5G). Interestingly, *FOXP3* was also correlated with the follicular T helper-associated chemokine *CXCL13*, which is expressed by exhausted CD8 T cells. Targeting *CXCL13* signaling to the associated recruitment receptor *CXCR3* on Tregs (Groom and Luster, 2011, Jenh et al., 2001) may therefore represent a therapeutic opportunity (Figure 4F).

Tumor infiltration or exclusion is another determinant of immune activity (Spranger et al., 2015). Fibroblasts, macrophages, and Tregs were most abundant at the tumor-stroma border, while CD8 T cells and neutrophils were largely excluded from the tumor, indicating that Treg positioning may prevent effector lymphocyte access to the tumor (Figures 5J, 5K, and S5H; STAR Methods). B cells, which have been shown to mediate either immune suppression or anti-tumor activity (Yuen et al., 2016), were the only cell type exhibiting preferential infiltration (Figure 5L). Spatial analysis of the TME at single-cell resolution therefore highlights localization patterns that may represent nodes for intervention in improving immune control.

### Mapping Cellular Crosstalk at Leading Edge Niches

**Figure 6:**
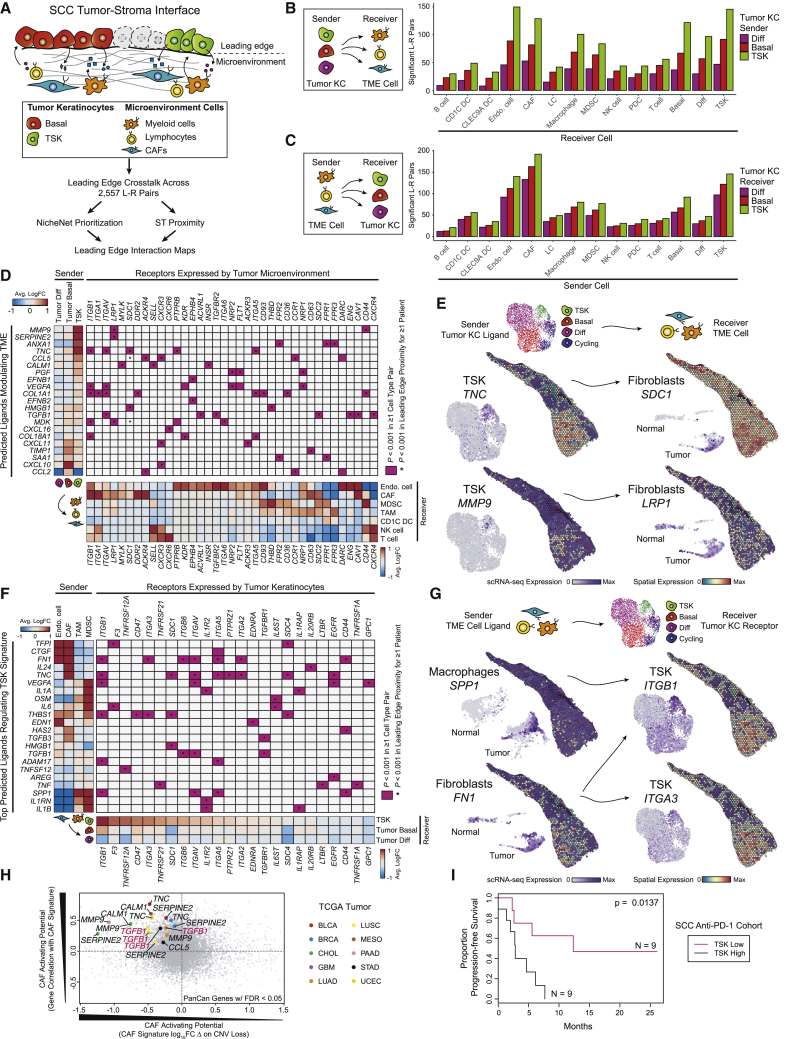
Cellular Crosstalk Landscape Associated with Leading Edge Niches (A) Schematic of combined scRNA-seq and ST ligand-receptor analysis of tumor keratinocyte (KC) subpopulations at the leading edge and TME cells. (B and C) Bar plots of significant ligand-receptor (L-R) pairs (p < 0.001, permutation test; STAR Methods) when tumors express ligands (B) or receptors (C) matched to cell types in scRNA-seq data. (D) Left, heatmap of scRNA-seq average log fold change (logFC) across normal and tumor KC subpopulations of NicheNet top predicted ligands expressed by tumor KCs that modulate TME cell types. Bottom, heatmap of scRNA-seq average logFC of ligand-matched receptors expressed by TME cell types. Middle, heatmap of significant ligand-receptor pairs between ≥1 tumor KC subpopulation and TME cell type pair in scRNA-seq. (E) UMAP scRNA-seq (N = 7 patients) and spatial feature plots (patient 4) of select ligands expressed by tumor KC subpopulations and cognate receptor expression by TME cell types. (F) Left, heatmap of scRNA-seq average logFC of NicheNet top predicted ligands expressed by TME cells that modulate the TSK signature. Bottom, heatmap of scRNA-seq average logFC of ligand-matched receptors across normal and tumor KC subpopulations (only tumor shown). Middle, heatmap of significant ligand-receptor pairs between ≥1 TME cell type and tumor KC subpopulation pair. ^∗^indicates spatial transcriptomic proximity p < 0.001 (permutation test; STAR Methods) in ≥1 patient in (D) and (F). Average logFCs in heatmaps from (D) and (F) were calculated using Wilcoxon rank-sum test. (G) UMAP scRNA-seq (N = 7 patients) and spatial feature plots (patient 4) of select ligands expressed by TME cell types and cognate receptor expression by tumor KC subpopulations. (H) Scatterplot of TCGA (31 cancer types) correlation values between single genes and CAF-signature expression (y axis) (FDR <0.05; STAR Methods) and effect of CNV loss on CAF-signature expression (x axis). TSK ligands are labeled and colored by cancer type. Known CAF activator *TGFB1* is highlighted in red. (I) Kaplan-Meier plot of progression-free survival after PD-1 inhibitor treatment of patient SCC (HNSC and LUSC) tumors exhibiting high and low TSK-associated gene expression (p value, chi-square test). See also Figure S6.

**Figure S6:**
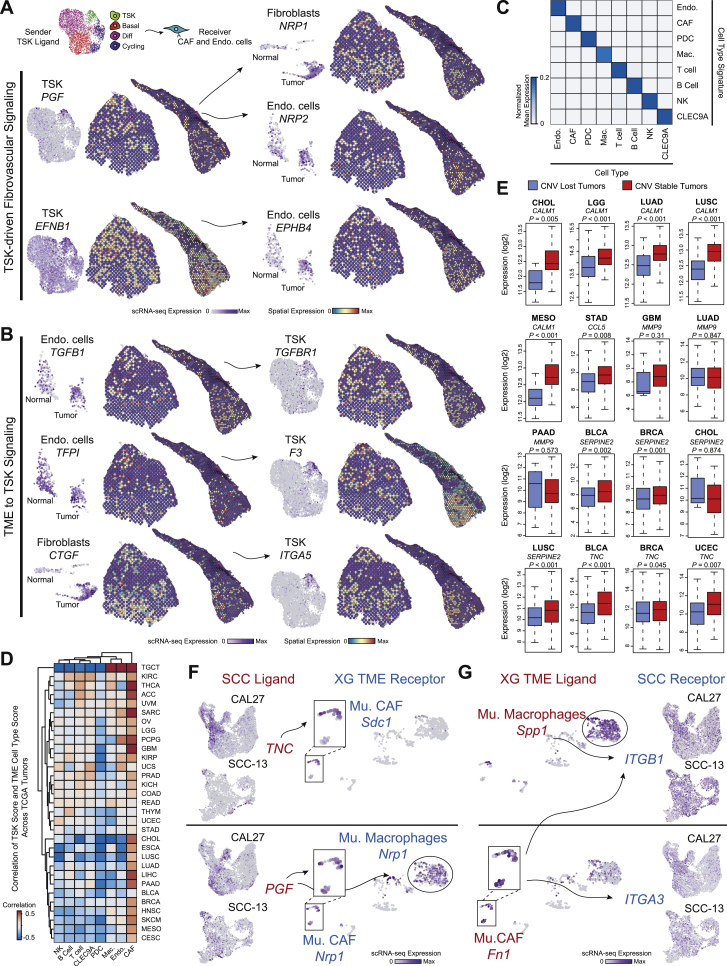
Ligand-Receptor Crosstalk in the TSK Niche, Related to Figure 6 (**A**) UMAP scRNA-seq (N = 7 patients) and spatial feature plots (N = 2 patients) of ligand-receptor and cell type pair expression highlighting TSK signaling to cancer-associated fibroblasts (CAFs) and endothelial cells. (**B**) UMAP scRNA-seq (N = 7 patients) and spatial feature plots (N = 2 patients) of ligand-receptor and cell type pair expression highlighting CAF or endothelial cell signaling to TSK. (**C**) Heatmap of average TME cell type signature expression across TME cell types in scRNA-seq data (**D**) Correlation heatmap of TME cell type signatures (columns) each correlated with the TSK signature across TCGA cancers (rows). (**E**) Boxplots showing RNA expression across TCGA cancers of specific genes in copy number variant (CNV) lost or stable tumors. P value was determined by Kolmogorov-Smirnov test. (**F**) UMAP feature plots of select ligands expressed by SCC cells and cognate receptors expressed by murine TME cell types. (**G**) UMAP feature plots of select ligands expressed by murine TME cell types and cognate receptors expressed by SCC cells.

Next, scRNA-seq and ST datasets were integrated to characterize signaling between adjacent tumor and TME cells at the leading edge (Figure 6A). Based on a database of ligand-receptor pairs (Ramilowski et al., 2015), TSKs participated in extensive autocrine and paracrine interactions (mostly with CAFs, endothelial cells, macrophages, and MDSCs) (Figures 6B and 6C; STAR Methods). We used NicheNet (Browaeys et al., 2019) and ST to prioritize tumor KC ligands predicted to modulate TME-specific cell-type signatures at the leading edge (STAR Methods). Consistent with a TSK-fibrovascular niche, prominent TSK signaling to CAFs was mediated by several TSK-CAF ligand-receptor pairs, including *MMP9*-*LRP1* and *TNC*-*SDC1* (Figures 6D, 6E, and S6A). Additionally, TSKs may modulate the endothelium through the ligand-receptor pairs *PGF*-*FLT1*, *PGF*-*NRP2*, and *EFNB1*-*EPHB4*.

Conversely, endothelial cells and CAFs prominently co-expressed NicheNet-predicted ligands such as *TFPI*, *FN1*, and *THBS1*, matching TSK receptors (Figures 6F, 6G, and S6B). Further supporting TSKs as an EMT-like population, NicheNet predicted that broadly expressed *TGFB1* and CAF-specific *TGFB3* regulate TSKs (David and Massagué, 2018). TSK receptors corresponding to additional ligands from TAMs and MDSCs included several integrins, including *ITGA3* and *ITGB1*, highlighting another pathway associated with EMT and epithelial tumor invasion (Margadant and Sonnenberg, 2010).

To address whether TSK cells were generally associated with an altered TME, we examined transcriptomic data from thousands of tumors across 31 solid tumor types in The Cancer Genome Atlas (TCGA). By analyzing TME gene signatures (from scRNA-seq), we observed a broad correlation between TSK and CAFs in various tumor types (Figures S6C and S6D). We then analyzed coupled tumor genomes and transcriptomes to prioritize genes functioning in the tumor to modulate the TME. Tumors with copy number variation (CNV) loss of a gene represent a “natural” genetic loss-of-function experiment, and, as expected, CNV loss was generally associated with lower expression (Figure S6E; STAR Methods). We next asked whether TSK ligands predicted to induce CAF genes exhibited both (1) reduced CAF gene expression in tumors with CNV loss of the ligand and (2) a correlation between ligand expression and CAF genes (Figure 6H). Indeed, several TSK ligands including *MMP9*, *TNC*, *SERPINE2*, *CALM1*, and the known CAF activator *TGFB1* appear to induce CAFs in human tumors (Kalluri, 2016, Löhr et al., 2001).

To extend these findings, we asked whether human clinical data could inform whether TSK cells suppress anti-tumor immunity. In a human clinical trial of PD-1 checkpoint inhibitor treatment of two types of squamous cell carcinoma (Prat et al., 2017), high expression of TSK-specific genes *ITGB1* and *PLAU* correlated with significantly reduced progression-free survival (p = 0.0137) (Figure 6I). While more work is required to understand whether this finding is due to intrinsic resistance of TSK cells or immune-modulating activity, it suggests that targeting this subpopulation may improve immunotherapy.

### Xenograft Tumors Recapitulate Cell Subpopulations in Spontaneous Human cSCC

**Figure 7:**
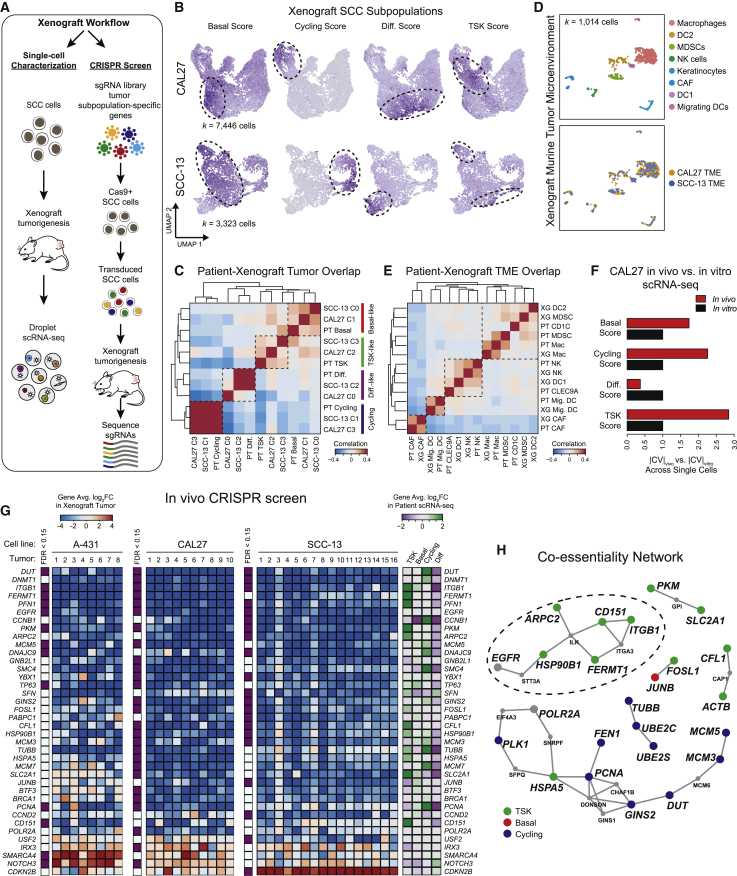
*In Vivo* CRISPR Analysis of Tumor Keratinocyte Vulnerabilities (A) Workflow of xenograft mouse models of human SCC cancer cells and CRISPR screen of tumor keratinocyte subpopulation-specific genes. (B) UMAP feature plots of signature scores derived from patient scRNA-seq data for basal, cycling, differentiating, and TSK scores in xenograft tumor cells. High-scoring subpopulations of cells are highlighted by dotted circle. (C) Differentially expressed gene (DEG) overlap correlation matrix across patient tumor subpopulations and unbiased clusters of xenografted SCC cell lines (STAR Methods). (D) UMAP of murine TME cells isolated from xenograft tumors. Top, labeled by cell type. Bottom, labeled by xenografted SCC cell line. (E) DEG overlap correlation matrix across patient and murine TME cell types demonstrating conserved cell-type expression programs across patient and xenograft TME cells. (F) Ratio of absolute coefficient of variation for subpopulation signatures between xenografted CAL27 cells and *in vitro* cultured CAL27 cells. (G) Left, heatmaps of mean log fold-change of sgRNAs targeting depleted and enriched genes in SCC cell lines (STAR Methods). All genes were significant by false discovery rate (FDR from STARS algorithm) <0.05 in ≥1 cell line. Right, heatmap of gene expression in patient scRNA-seq. (H) Co-essentiality network plot of depleted genes with STARS FDR <0.10 in ≥1 xenograft tumor line. Larger nodes indicate inclusion in screen and are colored by subpopulation designation while smaller node and font labels indicate connecting genes not included in screen. Gray larger nodes indicate broadly expressed genes. Edges represent a co-essential relationship at a previously determined FDR <0.10 (Wainberg et al., 2019). See also Figure S7.

**Figure S7:**
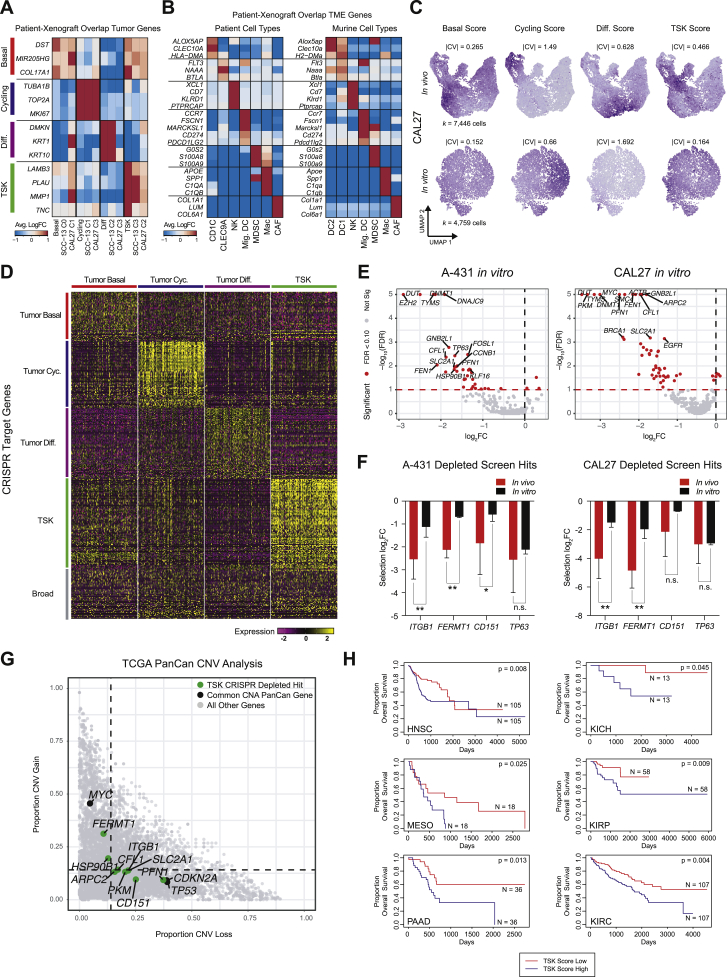
Analysis of *In Vivo* and *In Vitro* CRISPR Screen, Related to Figure 7 (**A**) Heatmap displaying average log fold-change of each tumor subpopulation-identifying gene relative to other subpopulations in corresponding patient sample or SCC cell line. (**B**) Heatmap displaying average log fold-change of each TME cell type-identifying gene relative to other cell types in either patient tumors or murine xenografts. (**C**) UMAP feature plots displaying patient-derived tumor subpopulation signatures for SCC cells harvested from xenograft tumors or *in vitro* culture. (**D**) Heatmap representing expression of genes targeted in CRISPR screen. Genes are grouped as tumor subpopulation-specific or broadly expressed (STAR Methods). (**E**) Volcano plots representing STARS false discovery rates (FDRs) and log2 fold-change (log_2_FC) values for each *in vitro* xenograft screen. Red dotted lines represent FDR 0.10. (**F**) Log2 fold-change (log_2_FC) values of select genes from CRISPR screens *in vivo* versus *in vitro*. Error bars represent standard deviation across tumor or cell culture biological replicates. Significance determined by permutation (STAR Methods). (^∗∗^: Permutation FDR < 0.05, ^∗^: Permutation FDR < 0.1). (**G**) Comparison of mean CNV gain and loss proportions across 31 TCGA tumor types for all genes. TSK-expressed CRISPR hits and control recurrently altered genes are highlighted. (**H**) Kaplan-Meier plots for TCGA tumor types with patients stratified by expression of TSK signature. P value derived from chi-square test.

Functional assessment of tumor subpopulation genes requires a representative experimental model of human cSCC. To address this, we generated tumors from human SCC lines in immune compromised mice and performed scRNA-seq (Figure 7A). Signature scoring and unbiased clustering of xenografted SCC cells supported the recapitulation of each of the four patient-derived subpopulations (Figures 7B, 7C, and S7A). Comparison of analogous TME cell types from patient and xenograft data also revealed high overlap of shared markers between human and mouse counterparts, with subtle differences among a few myeloid cell types in xenografts (Figures 7D, 7E, and S7B). *PD-L1/2* was expressed on migrating murine DCs, supporting a cross-species conserved and coupled regulatory-activation program in DCs, similar to recent work (Maier et al., 2020). Furthermore, heterotypic ligand-receptor pairs in SCC and TME murine cells were present in xenografts, indicating potential conservation of immune and stromal cell recruitment and crosstalk (Figures S6F and S6G). Analysis of SCC cell lines cultured *in vitro* using patient tumor subpopulation signatures revealed that *in vitro* cells exhibited far less heterogeneity in basal, cycling, and TSK scores, consistent with a TME requirement for emergence of tumor cell populations (Figures 7F and S7C). We therefore conclude that the xenograft model recapitulates the key features of cSCC ITH.

### *In Vivo* CRISPR Screen of Tumor KC Subpopulation Genes

We next assessed the functional roles of tumor KC subpopulations by performing CRISPR/Cas9 screens in three SCC xenografted tumor lines with 334 genes enriched in each of the four cSCC tumor subpopulations (Figures 7A and S7D; STAR Methods). We used the STARS algorithm (Doench et al., 2016) to derive 38 hits, 18 of which were shared across at least two cell lines (Figure 7F; STAR Methods). As expected, genes involved in cell-cycle and nucleotide metabolism, such as *DUT* and *MCM5*, were required for tumor growth. Specific TFs were also essential, including *TP63*, the epidermal master regulator and cSCC oncogene (Keyes et al., 2011), as well as the AP1 family TFs, *FOSL1* and *JUNB*. *FOSL1* is selectively expressed in TSK cells and was a predicted TSK regulator by SCENIC (Figure S2G; Table S2). Notably, genes important for tumor growth were biased for expression in TSK, basal, and cycling cells.

To explore functional groupings of hits, we leveraged co-essentiality analysis, which scores gene pairs for patterns of depletion across a collection of CRISPR screens in hundreds of cancer cell lines (Pan et al., 2018, Tsherniak et al., 2017) (Figure 7G; STAR Methods). Genes involved in the cell cycle that were specific to tumor cycling KCs formed the largest network, centered around *PCNA* and *GINS2*. The largest TSK-specific network consisted of *ITGB1*, *FERMT1*, *CD151*, *ARPC2*, and *HSP90B1*. The protein products of *ITGB1*, *CD151*, and *FERMT1* physically interact at the cell surface to mediate integrin signaling (Calderwood et al., 2013, Hemler, 2005). We reasoned that if integrin signaling mediates communication with the TME, the associated genes should display greater dependence in tumorigenesis *in vivo* than in the cell culture *in vitro*. To test this, we performed an identical CRISPR screen *in vitro*, where *ITGB1*, *FERMT1*, and *CD151* proved less important for tumor growth than *in vivo* (Figures S7E and S7F). These findings are consistent with integrins transmitting pro-tumorigenic signals from the TME. We further assessed the role of TSK-associated essential genes in TCGA. Specifically, we observed that *FERMT1* is recurrently amplified across many subtypes of cancer, suggesting a broadly pro-tumorigenic role (Figure S7G). Tumors from TCGA with high TSK gene expression were associated with worse overall survival across six epithelial cancers (Figure S7H), consistent with this subpopulation’s role in malignant behavior. In sum, *in vivo* xenograft screens coupled with TCGA analysis highlight critical genes that may control subpopulation-specific pro-tumorigenic functions.

## Discussion

Here, we used scRNA-seq to construct a single-cell transcriptomic atlas of cell subpopulations within cSCC and integrated spatial profiling with ST and MIBI to assess the niches within which these cells reside. The integrated spatial profiling approach helped overcome limitations of any individual method. Our experimental design choices to perform ST (50–100 μm resolution, 7 × 7 mm sections) and MIBI (cellular resolution, 800 × 800 μm for 17 FOVs, 2 × 2mm for one FOV) balanced tissue availability, technical constraints, and the biological questions motivating our study. Future efforts may achieve the scale of ST (essentially an entire biopsy) with the single-cell resolution of MIBI and facilitate detailed interrogation of structures beyond the leading edge.

The differentiation hierarchy in tumor KCs is reminiscent of developmental states in oligodendroglioma and expression of differentiation genes in subpopulations of oropharyngeal carcinomas (Puram et al., 2017, Tirosh et al., 2016b). We extended this concept by determining that several tumor subpopulations were strikingly similar to normal counterparts, while one subpopulation induced widespread tumor-specific genes. Single-cell studies of breast and lung tumors including non-malignant tissue found a majority of tumor cells did not resemble normal counterparts, possibly indicating a distinct paradigm in those tissues (Lambrechts et al., 2018, Wagner et al., 2019). The identification of TSK-enriched genes implicated in EMT as well as intercellular communication with CAFs and endothelial cells raises the possibility that TSK cells induce the fibroblast and endothelial states shown to contribute to tumor progression, immunosuppression, and heterogeneity (Costa et al., 2018, Hu et al., 2012, Wagenblast et al., 2015).

*In vivo* CRISPR screens highlighted a TSK-specific co-essential gene network. *ITGB1* and *CD151* are known to be pro-tumorigenic by enhancing cell migration and stromal invasion (Reuter et al., 2009, White et al., 2004, Yang et al., 2008, Zijlstra et al., 2008). *FERMT1* (also known as kindlin-1) directly binds the cytoplasmic tail of β1 integrin to enable signaling and mediate cell spreading and controls murine cutaneous stem cell homeostasis (Kloeker et al., 2004, Larjava et al., 2008, Rognoni et al., 2014). Further examination of this network may provide subpopulation-directed therapeutic avenues in cSCC.

Our results also begin to inform the clinical management of carcinomas. Patients with a high expression of the TSK markers *ITGB1* and *PLAU* exhibited significantly lower progression-free survival after treatment with PD-1 checkpoint inhibitors. More work is needed to understand whether this finding is due to immunosuppressive activity of TSKs or an intrinsic resistance to immune attack. The latter possibility is consistent with TSKs resembling tumor-initiating stem cells better equipped to survive adoptive cytotoxic T cell therapy in a mouse model of cSCC (Miao et al., 2019). While the link between EMT and drug resistance is well established (Shibue and Weinberg, 2017), our CRISPR screen provided functional evidence for several integrin-related genes, which may represent additional druggable vulnerabilities. Along those lines, the observed co-essentiality coupling of *EGFR*, which is broadly expressed in tumors, to the integrin signaling network nominates combined inhibition of EGFR and integrin signaling for treatment of advanced cSCC (Figure 7G) (Maubec et al., 2011). Beyond the findings described here, we anticipate this collection of data to serve as a resource informing the basic understanding of tumor development, the relationship between tumor and the TME, and therapies targeting various nodes of this complex ecosystem.

## STAR★Methods

### Key Resources Table

**Table:** 

REAGENT or RESOURCE	SOURCE	IDENTIFIER
**Antibodies**		

Anti-Human COL17A1	Sigma-Aldrich	Cat#HPA043673; RRID:AB_10960893
Anti-Human CD45 V450 (clone HI30)	BD Biosciences	Cat#560367; RRID:AB_1645573
Anti-Human FLAG (clone M2)	Millipore Sigma	Cat#F3165; RRID:AB_259529
MIBI Panel Antibodies	This paper	Table S5

**Bacterial and Virus Strains**		

Stellar Competent Cells	Takara Bio	Cat#636763

**Biological Samples**		

Human primary cSCC and normal skin samples	Stanford University School of Medicine	See Figure S1A for details

**Chemicals, Peptides, and Recombinant Proteins**		

Corning Matrigel Basement Membrane Matrix	Corning	Cat#354234
Tissue-TEK OCT Compound	Sakura Finetek	Cat#4583
Formalin solution, neutral buffered, 10%	Sigma-Aldrich	Cat#HT5011-1CS
Trypsin-EDTA 0.25%	GIBCO	*Cat#25200114*
DNase I	Worthington	Cat#LS002007
Collagenase I	GIBCO	*Cat#17100017*

**Critical Commercial Assays**		

Chromium Single Cell 3′ Gene Expression Solution v2	10X Genomics	Cat#PN-120237
MiSeq Reagent Kit v3 (150 cycle)	Illumina	Cat#MS-102-3001
KAPA Library Quantification Kit	Roche	Cat#07960298001
DNeasy Blood and Tissue Kit	QIAGEN	Cat#69504
Visium Spatial Gene Expression Solution v1	10X Genomics	Cat#PN-1000184
Visium Spatial Tissue Optimization Slide & Reagent Kit	10X Genomics	Cat#PN-1000193

**Deposited Data**		

Raw and analyzed sequencing data	This paper	GSE144240
MIBI data: https://mibi-share.ionpath.com	This paper	N/A

**Experimental Models: Cell Lines**		

Human: A-431	ATCC	Cat#CRL-1555; RRID:CVCL_0037
Human: CAL27	ATCC	Cat#CRL-2095; RRID:CVCL_1107
Human: SCC-13	Harvard Human Skin Disease Resource Center, James Rheinwald Lab	RRID:CVCL_4029
Human: HEK293T Lenti-X	Clonetech	Cat#632180
		

**Experimental Models: Organisms/Strains**		

Mouse: SCID Hairless Outbred Mice, Crl:SHO*-Prkdc*^*scid*^*Hr*^*hr*^	Charles River Laboratories	Cat#474
Mouse: NOD.Cg-*Prkdc*^*scid*^*Il2rg*^*tm1Wjl*^/SzJ Mice	The Jackson Laboratories	Cat#005557; RRID:IMSR_JAX:005557

**Oligonucleotides**		

CRISPR Screen Guide Oligos	This paper	Table S6
Primers for sgRNA sequencing for CRISPR screens	This paper	Table S6

**Recombinant DNA**		

pLentiguide	Rubin et al., 2019	Addgene: 117986
pLex_Cas9	Rubin et al., 2019	Addgene: 117987

**Software and Algorithms**		

Cell Ranger versions 2.1/3.0	10X Genomics	https://www.10xgenomics.com/; RRID:SCR_017344
ST Pipeline version 1.7.2	Navarro et al., 2017	https://github.com/SpatialTranscriptomicsResearch/st_pipeline
ST Spot Detector	Wong et al., 2018	https://github.com/SpatialTranscriptomicsResearch/st_spot_detector
Space Ranger version 1.0.0	10X Genomics	https://10xgenomics.com
Seurat versions 2.3.0/3.1.3	Butler et al., 2018	https://satijalab.org/seurat/; RRID:SCR_016341
SCENIC version 1.1.2.2	Aibar et al., 2017	https://github.com/aertslab/SCENIC; RRID:SCR_017247
NicheNet	Browaeys et al., 2019	https://github.com/saeyslab/nichenetr
STUtility version 1.0.0	This paper	https://ludvigla.github.io/STUtility_web_site/index.html
MIBIAnalysis	Keren et al., 2018	https://github.com/lkeren/MIBIAnalysis
FlowSOM	Van Gassen et al., 2015	https://github.com/SofieVG/FlowSOM; RRID:SCR_016899)
DeepCell	Van Valen et al., 2016	https://github.com/vanvalenlab/deepcell-tf

### Resource Availability

#### Lead Contact

Further information and requests for resources and reagents should be directed to and will be fulfilled by the Lead Contact, Paul A. Khavari (khavari@stanford.edu).

#### Materials Availability

This study did not generate new unique reagents.

#### Data and Code Availability

The accession number for the raw and processed sequencing data reported in this paper is Gene Expression Omnibus (GEO): GSE144240 (https://www.ncbi.nlm.nih.gov/geo/). All analysis scripts are available on reasonable request. Public access to the MIBI data described here is freely available via the public instance of the MIBItracker (Ionpath Inc) at https://mibi-share.ionpath.com/. A full description for how to use the MIBItracker is available here: https://storage.googleapis.com/mibitracker-static/docs/MIBItrackerUserGuide.pdf

### Experimental Model and Subject Details

#### Human Patient Samples

Cutaneous SCCs and patient-matched normal adjacent skin samples were collected under a protocol approved by the Institutional Review Board at Stanford University Medical Center (Protocol #21750). Individuals donating fresh surgical tissue provided informed consent. All diagnoses were verified by histological review by a board-certified dermatopathologist. For tumors of adequate size, small fragments of each tumor were snap frozen in optical cutting tissue (OCT) compound (Tissue-Tek, Sakura Finetek USA Inc., Torrance, CA) and/or fixed in 10% formalin (Sigma-Aldrich, St. Louis, MO).

#### Cell Lines

The human SCC cell line SCC-13 (a generous gift from J.G. Rheinwald, Dana-Farber/ Harvard Cancer Center) was cultured in KSFM supplemented with 25 μg/ml BPE, 0.2 ng/ml EGF (GIBCO, Thermofisher Scientific, Waltham, MA) and 0.3 mM CaCl2 (Sigma-Aldrich) (hereinafter SCC-13 media). The human SCC cell lines A-431 and CAL27 were obtained from the American Type Culture Collection and grown in DMEM (GIBCO) supplemented with 10% FBS (HyClone, GE Healthcare, Chicago, IL). All cells were grown at 37°C in a humidified chamber with 5% CO_2_. All cell lines were negative for mycoplasma with MycoAlert (Lonza, Basel, Switzerland) immediately before use.

#### Mice

SCID Hairless Outbred (SHO, Crl:SHO*-Prkdc*^*scid*^*Hr*^*hr*^*,* Charles River) mice and NOD.Cg-*Prkdc*^*scid*^ *Il2rg*^*tm1Wjl*^/SzJ (Jackson Laboratory) were all female and 8-9 weeks in age at the time of injection for xenograft tumor experiments. All mouse husbandry and experimental procedures were performed in accordance and compliance with policies approved by the Stanford University Administrative Panel on Laboratory Animal Care (Protocol #9863). Mice were housed under standard conditions with food and water provided *ad libitum* and maintained on a 12-h light, 12-h dark cycle.

### Method Details

#### Tissue Dissociation

Fresh tumor specimens and normal skin were minced with a scalpel in serum-free DMEM (GIBCO) with DNase I (0.2 mg/mL, Worthington, Inc., Columbus, OH) to pieces < 1 mm^3^ and placed in 5mL of 0.25% trypsin-EDTA (GIBCO) at 37°C for 30 minutes with trituration with a wide-bore pipet tip every 10 minutes. For normal skin, subcutaneous fat was removed with surgical scissors prior to mincing and trypsin-EDTA incubation. After 30 minutes, trypsin-EDTA was quenched with DMEM supplemented with 10% FBS and cells were filtered through a 70 μm filter (BD Falcon). Dissociated cells were pelleted and washed once with cold serum-free DMEM with DNase I and viability > 90% was confirmed with trypan blue staining (Invitrogen), after which cells were immediately cryopreserved in 90% SCC-13 media with 10% DMSO (Sigma-Aldrich). All steps other than trypsin incubation were completed at 4°C or on ice. For patients 3 and 8, samples were incubated in 0.25% trypsin-EDTA and type I collagenase 5mg/mL (GIBCO) for 30 minutes (instead of trypsin-EDTA 0.25% alone for other patients), which led to recovery of higher numbers of T cells in normal skin samples from these patients (Figure 1F). However, we did not observe major differences in downstream processing of cells recovered from collagenase dissociation (Figure S1B).

#### Sorting of Patient Samples

On the day of scRNA-seq droplet capture, cells were thawed in at least 5X dilution volume of SCC-13 media to wash off residual DMSO in cryopreservation media. Cells were pelleted and resuspended in cold phosphate buffered saline (PBS) (GIBCO) with 2% FBS and 2mM EDTA (Invitrogen, Carlsbad, CA). Cells were stained with propidium iodide (Sigma-Aldrich) prior to sorting on a BD FACS Aria II. For patients 1 and 8, cells were stained with anti-CD45 antibody conjugated to V450 (clone HI30, 560367, BD Biosciences) to separate immune cells from non-immune cells. For patient 1, only CD45-negative cells were sequenced while for patient 8, both CD45+ and CD45- fractions were sequenced, neither of which significantly affected downstream data processing. Sorted cells were pelleted and resuspended in PBS with 2% FBS and 2mM EDTA prior to scRNA-seq droplet capture.

#### Single-cell RNA-sequencing

scRNA-seq was performed immediately after FACS sorting by the Stanford Functional Genomics Facility (SFGF) with the 10X Chromium 3′ v2 kit (10X Genomics, Pleasanton, CA) following the manufacturer’s protocol. Target number of captured cells ranged from 2,000 to 10,000 cells. Sequencing libraries were prepared per manufacturer’s protocol. Sequencing was performed on a HiSeq 4000 (Illumina, Inc., San Diego, CA) at a median depth of 115,216 reads/cell (Table S1). Raw sequencing data was processed with the cellranger pipeline (version 2.1.0, 10X Genomics) and mapped to the hg19 reference genome to generate matrices of gene counts by cell barcodes.

#### Whole exome sequencing: experimental

Approximately 10-12 8 μm sections of fresh-frozen tissue in OCT were dissolved in PBS to remove OCT. The tissue was pelleted and used as input for the DNeasy Blood and Tissue Kit (QIAGEN, Hilden, Germany) to extract genomic DNA. The Agilent V6 whole exome capture kit (60 Mbp) was used and the resulting libraries were sequenced on the BGISEQ-500 instrument (BGI Group, Shenzhen, China) using paired-end 100bp reads to achieve approximately 100x coverage for each tumor sample and 50x coverage for each normal skin sample.

#### Spatial Transcriptomics

##### Slide preparation

Spatial Transcriptomics slides were printed with six identical 6.3 × 6.7 mm capture areas, each with 1,934 clusters (spots) of barcoded primers (10X Genomics). The primers are attached to the slide by the 5′ end and contain a cleavage site, a T7 promoter region, a partial read 1 Illumina handle, a spot-unique spatial barcode, a unique molecular identifier (UMI), and a 20(T)VN. The spots have a diameter of ∼110 μm and are arranged in a centered rectangular lattice pattern so that each spot has four surrounding spots with a center-to-center distance of 150 μm, forming a 310 μm x 325 μm rectangle (Figure S3A).

##### Tissue optimization

The Spatial Transcriptomics (ST) protocol was optimized for cSCC tissue according to recommendations (Salmén et al., 2018b). In short, changes were made in the staining procedure by excluding isopropanol, decreasing the incubation time of hematoxylin and bluing buffer, as well as increasing eosin concentration. Moreover, the optimal incubation time for permeabilization was established, and the previously described one-step protocol for tissue removal was altered by using a higher proteinase K:PKD buffer ratio (described in further detail in following sections). Once optimal conditions had been established, three cryosections per patient were cut at 10 μm thickness onto spatial slides and processed immediately.

##### Fixation, staining and imaging

Sectioned slides were incubated at 37°C for 1 min., fixed in 3.7%–3.8% formaldehyde (Sigma-Aldrich) in PBS (Medicago) for 10 min, and then washed in 1x PBS (Medicago).

For staining, sections were incubated in Mayer’s hematoxylin (Dako, Agilent, Santa Clara, CA) for 4 min, bluing buffer (Dako) for 30 s, and Eosin (Sigma-Aldrich) diluted 1:5 in Tris-base (0.45M Tris, 0.5M acetic acid, pH 6.0) for 30 s. The slides were washed in RNase and DNase free water after each of the staining steps.

After air-drying, the slides were mounted with 85% glycerol (Merck Millipore, Burlington, MA) and coverslips (Menzel-Gläser). Bright-field (BF) images were taken at 20x magnification using Metafer Slide Scanning platform (MetaSystems). Raw images were stitched with VSlide software (MetaSystems). The coverslip and glycerol were removed after imaging by immersing slides in RNase and DNase free water.

##### Tissue permeabilization

The slides were inserted into slide cassettes to separate the tissue sections into individual reaction chambers (hereinafter wells). For pre-permeabilization, sections were incubated at 37°C for 20 min with 0.5 U/μl collagenase (ThermoFisher) and 0.2 μg/μl BSA (NEB, Ipswich, MA) in HBSS buffer (ThermoFisher). Wells were washed with 0.1 × SSC (Sigma-Aldrich), after which permeabilization was conducted at 37°C for 7 min in 0.1% pepsin (Sigma-Aldrich) dissolved in 0.1M HCl (Sigma-Aldrich). After incubation, the pepsin solution was removed and wells washed with 0.1 × SSC.

##### Reverse transcription, spatial library preparation and sequencing

Reverse transcription (RT) was conducted as previously described (Salmén et al., 2018b). After RT, wells were washed with 0.1 × SSC and incubated at 56°C with interval shaking for 1.5 h with a tissue removal mix of Proteinase K (QIAGEN) and PKD buffer (QIAGEN, pH 7.5) at a 1:1 ratio. The spatially barcoded cDNA was enzymatically released as previously described (Salmén et al., 2018b).

Supernatants containing released cDNA were collected and transferred to 96-well plates for ST library preparation with an automated MBS 8000 system (Jemt et al., 2016). In short, second-strand cDNA synthesis was followed by *in vitro* transcription, adaptor ligation, and a second RT. Sequencing handles and indexes were added in an indexing PCR and the finished libraries were purified and quantified as previously described (Ståhl et al., 2016).

The libraries were sequenced on the Illumina NextSeq platform with 31 bases from read 1 and 46 from read 2 and 47 to 221 million raw reads were generated per sample.

##### Spot visualization and image alignment

The spot staining and imaging procedure was described previously (Salmén et al., 2018b). In short, primer spots were stained by hybridization of fluorescently labeled probes and imaged on the Metafer Slide Scanning platform. The resulting spot image was loaded into the web-based ST Spot Detector tool (Wong et al., 2018) along with the previously obtained BF tissue image of the same area. The two images were aligned and the built-in tissue recognition tool was used to extract spots covered by tissue.

##### Spatial Transcriptomics raw data processing

Raw sequencing data was processed using the open source ST Pipeline v1.7.2 (Navarro et al., 2017) with the GRCh38 v86 genome assembly as reference and corresponding GENCODE annotation file (version 25). The ST pipeline was executed with two-pass mode enabled for alignment and discarding reads in which the UMI had at least 6 low quality bases. The count matrices were filtered to keep only protein-coding, long non-coding, and antisense genes, and Ensembl IDs were replaced by HGNC symbols.

#### Visium Spatial Gene Expression

##### Tissue optimization (TO)

The Visium Spatial Tissue Optimization Slide & Reagent kit (10X Genomics) was used to optimize permeabilization conditions for cSCC tissue. The manufacturer’s protocol was followed except for a decrease in Hematoxylin staining time to 4 minutes.

##### Preparation of Visium Sequencing Libraries

The Visium Spatial Gene Expression Slide & Reagent kit (10X Genomics) was used to generate sequencing libraries. Two sections per patient were cut at 10 μm thickness and mounted onto Visium slide capture areas. Sequencing libraries were prepared following the manufacturer’s protocol, again with the Hematoxylin staining time adapted to 4 minutes. After tissue staining, bright-field images were taken as described in the Spatial Transcriptomics procedure. Tissue permeabilization was performed for 14 minutes, as established in the TO assay.

##### Sequencing and data processing

The libraries were sequenced on a NextSeq (Illumina), with 28 bases from read 1 and 120 from read 2, and at a depth of 84-116 million reads per sample. Following demultiplexing of bcl files, the read 2 fastq files were trimmed using Cutadapt (Martin, 2011) (Martin, 2011) to remove full-length or truncated template switch oligo (TSO) sequences from the 5′ end and polyA homopolymers from the 3′ end. The TSO sequence (AAGCAGTGGTATCAACGCAGAGTACATGGG) was used as a non-internal 5′ adaptor with a minimum overlap of 5, meaning that partial matches (up to 5 base pairs) or intact TSO sequences were removed from the 5′ end of read 2. The error tolerance was set to 0.1 for the TSO trimming to allow for a maximum of 3 errors. For the 3′ homopolymer trimming, a sequence of 10 As was used as a regular 3′ adaptor to remove potential polyA tail products regardless of its position in the read, also with a minimum overlap of 5 base pairs. The trimmed data was processed with the spaceranger pipeline (version 1.0.0, 10X Genomics) and mapped to the GRCH38 v93 genome assembly.

#### MIBI Image Acquisition

##### Gold Slide Preparation

Slide preparation was performed at the Stanford Nano Shared Facility (SNSF) as previously described (Keren et al., 2018). In short, Superfrost glass slides (Thermofisher Scientific) were soaked in 0.1% SDS and rinsed with distilled water, and subsequently acetone (Thermofisher Scientific). Slides were then coated with 30nM of tantalum and 100nM of gold. Slides were then silanized in acetone (Thermofisher Scientific) before treatment with Vectabond (Vector Labs, Inc., Burlingame, CA). Slides were baked for 70°C for 30 minutes and stored at room temperature before use.

##### Antibody conjugation

A summary of antibodies, reporter isotopes, and concentrations can be found in Table S5. Metal conjugated primary antibodies were prepared as described previously (Han et al., 2018). Following labeling, antibodies were diluted in Candor PBS Antibody Stabilization solution (Candor Bioscience GmbH, Wangen, Germany) at least by 15% w/v, to a concentration no lower than 0.2 mg/mL and stored long-term at 4°C.

##### Staining

Patient samples were fixed for 24 hours in 10% formalin (Sigma-Aldrich) and then transferred to 70% ethanol for long-term storage. Fixed tissue was embedded in paraffin by Histo-Tec laboratories (Hayward, CA). Tissue sections (4 μm thick) were cut from FFPE tissue blocks using a microtome at the Stanford University Histology Service Center and mounted onto gold slides for MIBI or glass slides for H&E/IHC. Slide-tissue sections were baked at 70°C for 1 hour and subsequently soaked in xylene (Thermofisher Scientific) for 2 × 10min. Tissue sections were then rehydrated with successive washes of xylene (3x) followed by ethanol at 100% (2x), 95% (2x), 80% (1x), 70% (1x), and distilled water (3x). Washes were performed using a Leica ST4020 Linear Stainer (Leica Biosystems, Wetzlar, Germany) programmed to 3 dips per wash for 180 s each. The sections were then immersed in epitope retrieval buffer (Target Retrieval Solution, pH 9, DAKO Agilent, Santa Clara, CA) and incubated at 97°C for 10 min and cooled down to 65°C using the Lab vision PT module (Thermofisher Scientific), before further cooling to room temperature. Slides were then washed with a wash buffer made with 1X TBS IHC Tween buffer (Cell Marque, Rocklin, CA) containing 0.1% (w/v) BSA (Thermofisher Scientific). Slides were then washed for 5 min in wash buffer with slow shaking on an orbital shaker 60rpm. Slides were then blocked for 1h with 2% (v/v) donkey serum (Sigma-Aldrich), 0.1% Triton X-100 (Sigma-Aldrich) and 0.05% Sodium Azide (Sigma-Aldrich) diluted in 1X TBS IHC wash buffer (Cell Marque). H&E staining, anti-CD163 (EDHu-1, Novus Biologicals) staining, and anti-COL17A1 (HPA043673, Sigma-Aldrich) staining were performed on glass slides for regional annotation, including identification of leading edge and stromal macrophages as a surrogate for TME inflammation. Metal-conjugated antibody mix (See Table S5) was prepared in 3% (v/v) donkey serum in 1X TBS IHC wash buffer and filtered using a 0.1um hydrophilic PVDG centrifugal filter (EMD Millipore). This antibody mix was incubated with slides overnight at 4°C in a humid chamber. After the overnight incubation, slides were washed twice on an orbital shaker for 5 min each at 60rpm in 1X wash buffer as above. Cells were then fixed for 10 min in a diluted glutaraldehyde solution 2% (Electron Microscopy Sciences, Hatfield, PA) in 1X PBS-low barium. Slides were then rinsed briefly in 1X PBS-low barium. Tissue sections were then dehydrated with successive washes of fresh Tris 0.1 M (pH 7.5) (3x), distilled water (3x), and ethanol at 70% (1x), 80%(1x), 95% (2x), and 100% (2x). Slides were immediately dried in a vacuum chamber for at least 1 h prior to imaging. All tissue staining was performed in parallel for all patient samples, using the same antibody mix to ensure reproducibility.

Quantitative imaging was performed using a custom designed and built MIBI-TOF mass spectrometer equipped with a duoplasmatron ion source (Ionpath Inc., Menlo Park, CA). This instrument was previously described in further detail (Keren et al., 2019). In brief, O^2+^ primary ions are focused onto the sample, and regions on the tissue are sequentially sputtered with the primary ion beam using a fly back raster pattern. Secondary ions were collected using a +70V sample bias tuned for preferential transmission of monoatomic ions relative to polyatomic organic species. The secondary ions then enter an orthogonal time of flight mass spectrometer with a mass range of 1-200 m/z+ and mass resolution of 1000 m/Δm operating at 100KHz repetition rate. Multiple TOF spectra for each pixel are summed and saved to a data file. The following MIBI-TOF imaging parameters were used in acquiring the imaging data:

Fields of View (FOVs) 1 – 17:

• Pixel dwell time: 7ms
• Image size: 800 μm x 800 μm at 2048 × 2048 pixels
• Probe size: 500nm
• Primary ion current: 3.4nA as measured via a faraday cup

FOV 18, comprising of 25 tiles at the following parameters:

• Pixel dwell time: 7ms
• Image size: 400 μm x 400 μm at 512 × 512 pixels
• Probe size: 300nm
• Primary ion current: 2.2nA as measured via a faraday cup

#### Xenograft Tumors

Two million SCC-13, CAL27, or A-431 cells were mixed with Matrigel (Corning, Corning, NY) and injected into the rear flanks of SCID Hairless Outbred (SHO) mice (Charles River). Tumors were allowed to grow for approximately 8-10 weeks, harvested, and dissociated into single-cell suspensions using the same protocol for patient samples as described above, including cryopreservation in 10% DMSO with appropriate cell culture media.

#### Sorting and scRNA-seq of Xenograft Tumors and *in vitro* Cells

Dissociated xenograft tumor single-cell suspensions were thawed in at least 5X dilution volume of appropriate culture media and resuspended in PBS with 2% FBS and 2mM EDTA. Viable cells were isolated by FACS sorting as described above and scRNA-seq, downstream library preparation, and sequencing was performed in the same manner as patient samples with the exception of the following: raw sequencing data was mapped to the combined mm10 and hg19 reference genomes to identify murine and cancer cell transcripts, respectively, with the 10X cellranger pipeline (version 2.1.0). For *in vitro* scRNA-seq, cultured CAL27 cells were washed with PBS twice after trypsinization, placed in PBS with 0.04% BSA, counted and directly loaded onto a 10X Chromium controller. Library preparation was performed with the Single Cell 3′ v3 kit (10X Genomics) per manufacturer’s protocol. Sequencing was performed on an Illumina HiSeq 4000 at a depth of 52,740 mean reads/cell. Raw sequencing data was mapped to the GrCH38 transcriptome with 10X cellranger version 3.0.2.

#### CRISPR Screen

##### CRISPR Guide Library Cloning

A 69nt guide oligo pool was ordered from GenScript with the following sequence: 5′-ATCTTGTGGAAAGGACGAAACACCG-[guide spacer sequence]-GTTTAAGAGCTATGCTGGAAACAG-3′. The oligo pool was PCR amplified for 15 cycles of 98°C for 10 s, 56°C for 30 s, and 72°C for 15 s using PrimeSTAR Max DNA Polymerase and Primer 1: ATCTTGTGGAAAGGACGAAACA and Primer 2: CTGTTTCCAGCATAGCTCTTAAAC. The amplified oligo pool was run on a 2% agarose gel and purified using the Zymoclean Gel Recovery kit. 16ng of the oligo pool was then assembled with 446ng Esp3I digested pLentiguide vector in a Gibson Assembly reaction (NEB). The reaction was transformed into Stellar competent cells and grown overnight in liquid culture with ampicillin selection. Following plasmid preparation using the Plasmid Plus Maxi Kit (QIAGEN), the library was sequenced on an MiSeq (Illumina, Inc.) to confirm guide representation.

##### *In vitro* and *in vivo* CRISPR Screens

SCC-13, CAL27, and A-431 cells were transduced with pLEX_Cas9 (Rubin et al., 2019; Addgene: 117987) lentivirus and selected with blasticidin HCl for three to four days to establish stably expressing Cas9 lines. pLEX_Cas9 lentivirus was produced in HEK293T Lenti-X cells (Clontech) seeded in 15cm plates at 80% confluence. Cells in each 15cm were transfected with 15 μg of p8.91, 15 μg of Cas9 vector, and 7.5 μg of pMDG in 2mL Opti-MEM (Thermo Fisher Scientific) and 70uL of Lipofectamine2000 (Thermo Fisher Scientific). Media was replaced after six hours. Supernatant was collected 48 hours after transfection, filtered through a 0.45 um PES membrane, and concentrated to 50X with Lenti-X concentrator (Takara). Cas9 expression was confirmed by anti-FLAG (F3165, Millipore Sigma) western blotting. Pooled guide lentivirus was titrated in each of the Cas9 expressing cell lines to achieve an MOI of 0.3. Following selection with puromycin for two to three days, three million cells were frozen at −80°C for the initial screen time point. Cells were cultured for three weeks for the *in vitro* CRISPR screen. For the *in vivo* CRISPR screen, two to four million cells in 100 μL were mixed with 50 μL Matrigel (Corning) and injected with 28G insulin syringes (Exel International) into the rear flanks of SHO mice (for A-431 and CAL27 lines) or NOD.Cg-*Prkdc*^*scid*^ *Il2rg*^*tm1Wjl*^/SzJ (Jackson Laboratory) for SCC-13 cells. All animals were female and 8-9 weeks in age at the time of injection. Resulting tumors were dissected after approximately two months. Tumors were minced and homogenized in an MP Biomedicals Benchtop Homogenizer (MP Biomedicals, Burlingame, CA) using MP Biomedicals lysing matrix A tubes. Genomic DNA was isolated from cells/tumors using the DNeasy Blood and Tissue Kit (QIAGEN).

##### CRISPR Sequencing Library Construction

Two rounds of PCR were used to generate CRISPR screen sequencing libraries (a full set of primers used is listed in Table S6). In PCR 1, guide sequences were amplified from genomic DNA using primers flanking the spacer sequence: Primer 3 ACACGACGCTCTTCCGATCTNNNNNNNNNTGTGGAAAGGACGAAACACC and Primer 4 GTGACTGGAGTTCAGACGTGTGCTCTTCCGATCGTAATACGGTTATCCACGCGG. Primer 3 contains nine degenerate nucleotides that are used to collapse PCR duplicates in downstream analysis. To maintain guide library complexity, 36 100uL PCR 1 reactions were set up, each containing 500ng of genomic DNA, PrimeSTAR Max DNA Polymerase, and primers 3 and 4. PCR 1 reactions were heated at 98°C for 10 s, 56°C for 15 s, and 72°C for 15 s for a total of 5 cycles. Reactions were concentrated using the DNA Clean and Concentrator-100 kit (Zymo, Irvine, CA). PCR 1 primers were then removed using 1.1X AMPure XP beads (Beckman Coulter Life Sciences).

A single 100 μL PCR 2 reaction was performed containing PrimeSTAR Max DNA Polymerase (Takara), the product from PCR 1, primers containing Illumina indexes and adaptor sequences, and SYBR green (Thermofisher Scientific). The same cycling conditions used in PCR 1 were followed in PCR 2. The reaction was monitored on a Stratagene MX3005P quantitative PCR (qPCR) machine and stopped in the linear phase of amplification, typically after 20-25 cycles. The PCR 2 product was gel purified on a 2% agarose gel. The resulting library was analyzed on a Bioanalyzer and quantitated by qPCR with the KAPA Library Quantification Kit (Roche) prior to library mixing and deep sequencing on an Illumina MiSeq.

### Quantification and Statistical Analysis

Statistical analyses were performed with GraphPad Prism version 8 (GraphPad Software, La Jolla, CA) or R versions 3.4.0 and 3.5.1. Parameters such as number of replicates, the number of independent experiments, measures of center, dispersion, and precision (mean ± SD or SEM), statistical test and significance, are reported in Figures and Figure Legends.

### Computational Methods

#### scRNA-seq Data Processing

Gene-barcode counts matrices were analyzed with the Seurat R package (version 2.3.0). Cells with < 200 genes detected and > 10% mitochondrial gene mapped reads were filtered from downstream analyses. All samples were merged with the AddSamples function into one Seurat object. The merged Seurat object was normalized and scaled by regressing out UMI count and percentage of mitochondrial genes. For dimensionality reduction, the most variable genes were determined using the FindVariableGenes function with parameters 0.0125 < mean of non-zero values < 3 and standard deviation > 0.5. Dimensionality reduction was then performed using PCA and UMAP plots were generated by the RunUMAP Seurat function (Seurat version 3.0.0) with the first 13-15 PCs as input, determined by visualizing the drop off in PC variance explained using the ElbowPlot function in Seurat.

#### Cell Type Annotation

In order to determine cell types, we combined unsupervised clustering and differential expression to compare top differentially expressed genes with cell type specific expression known from literature. Through this approach, we confidently identified broad categories among all cells, and further delineated cellular subtypes by isolating subsets (through *in silico* “gating”) of broadly defined cell types and re-analyzing with the same approach. For broad cell type annotation shown in Figure 1B, low-resolution clustering was performed using the FindClusters function with resolution 0.1 with the first 15 PCs to generate 12 clusters. Differential expression was performed using the FindAllMarkers function in Seurat with default parameters. Four of these clusters (clusters 0, 2, 3, and 5) highly expressed epithelial-associated genes such as *KRT5*, *KRT14*, *KRT1*, and *KRT10* and were therefore merged and inferred to be epithelial cells. Myeloid cells made up two clusters (clusters 1 and 4) and highly expressed *LYZ* and MHC class II genes (e.g., *HLA-DRB1*, *HLA-DRA*, and *HLA-DQB2*). Other clusters highly expressed markers specific for T cells (*CD3D*, *CD2*, and *CD7*; later determined to also include NK cells after re-clustering), fibroblasts (*COL1A1*, *COL1A2*, *LUM*), melanocytes (*MLANA*, *DCT*, *PMEL*), endothelial cells (*TFF3*, *CLDN5*, *VWF*), and B/plasma cells (*IGLL5*, *IGJ*, *MS4A1*, *CD79A*).

Myeloid cell subset annotations were determined by isolating their respective clusters, repeating dimensionality reduction and unsupervised clustering on these smaller groups of cells. For myeloid cell types, clustering at resolution 0.6 followed by differential expression demonstrated clusters high in the monocyte marker *CD14*, which were further re-clustered into macrophages (*CD163*, *CD68*) and MDSCs (*S100A8*, *S100A9*, *TREM1*). The remaining subsets were re-clustered into CD1C DCs (*CD1C*, *CLEC10A*), CLEC9A cells (*CLEC9A*, *CADM1*, *XCR1*), Langerhans cells (*CD207*, *CD1A*, *S100B*), AS DCs (*AXL*, *IGFBP5*, *PPP1R14A*), plasmacytoid DCs (*CLEC4C*, *IL3RA*), and migrating DCs (*CCR7*, *CCL19*) (Bronte et al., 2016, Kiss et al., 2018, Villani et al., 2017). Migrating DCs were further isolated, re-clustered into three groups, which were inferred to be CD1C DCs, CLEC9A DCs, and Langerhans cells based on similar expression to aforementioned markers.

T cell subset annotations were determined by further regressing patient identity using the ScaleData function in Seurat, and re-clustering the T cell cluster at higher resolution (2.0) to obtain 11 clusters. These clusters corresponded to the identities in Figure 1D and gene expression heatmap in Figure S4B, inferred from previous studies (Guo et al., 2018, Savas et al., 2018, Tirosh et al., 2016a, Zhang et al., 2018, Zheng et al., 2017). During all aforementioned re-clustering for mononuclear and T cell subsets, we also observed small clusters of cells co-expressing keratinocyte markers such as *KRT5* or *KRT1* (for both mononuclear and T cells), or mononuclear markers such as *LYZ* (for T cells), which we inferred to be doublets and removed from further analysis.

Cell cycle regression was performed using the CellCycleScoring function in Seurat to obtain S and G2/M phase scores for single cells, followed by the ScaleData function to regress out S and G2/M scores, and re-clustering to obtain the appropriate number of clusters. Proportion of cycling cells were obtained by calculating the number of previously identified cycling cells (pre-regression) that clustered with new clusters post-regression divided by the total number of cells in the post-regression clusters.

#### Keratinocyte Subpopulation Analysis

Keratinocytes were isolated from the epithelial clusters in the broad cell type UMAP (Figure 1B) and repeating dimensionality reduction with PCA and clustering at resolution 0.6. We identified putative pilosebaceous clusters based on expression of known hair follicle markers or transcription factors (*KRT15*, *LHX2*, *SOX9*) and prior mouse and human studies in addition to manual cross-referencing of the Human Protein Atlas (Joost et al., 2016, Purba et al., 2015, Uhlén et al., 2015) and removed these from further analysis. We separated normal keratinocytes from tumor keratinocytes based on sample origin and additionally removed a small number of cells coming from tumor samples that clustered with normal cells and vice versa. Normal keratinocytes were re-scaled with regression of patient identity and re-clustered into three clusters with representation from all patients (Figures 2A, 2B, and S2D).

Among the eight patients with overlapping clusters of tumor keratinocytes, we employed canonical correlation analysis (CCA) to align the patient data using the Seurat package (version 2.3.0). We recovered too few cells from Patient 3 to complete CCA, and these cells were removed from further analysis. For the cells from the remaining seven patients, we performed clustering (resolution 0.25) and UMAP with the first 13 CCA components in Seurat (version 3.1.3). Cell cycle proportion analysis was performed as described above for other subsets of cells. Upon cell cycle regression of normal keratinocytes, a small cluster of inferred cycling pilosebaceous keratinocytes emerged and was removed from additional analysis. Differential expression was performed using the FindAllMarkers function in Seurat with default parameters. Genes filtered for adjusted p value < 0.05 (Wilcoxon Rank Sum test) were included in gene marker overlap analysis (Figure 2D; Table S2). Gene ontology (GO) analysis was performed using DAVID v6.8 (Huang et al., 2009) on the top 200 differentially expressed genes (adjusted p value < 0.05 by Wilcoxon Rank Sum test). GO terms shown in Figure 2E are enrichment at false-discovery rate (FDR) < 0.15 in ≥ 1 subpopulation. Hallmark EMT signature scoring was performed using the AddModuleScore function in Seurat with default parameters. SCENIC analysis was performed on tumor keratinocytes with recommend parameters from tutorials available on the SCENIC Github page (https://github.com/aertslab/SCENIC) (Aibar et al., 2017).

#### Xenograft scRNA-seq analysis

Xenograft single-cell scoring was performed using the AddModuleScore function with default parameters in Seurat with the top 85-100 significantly differentially expressed genes in tumor keratinocyte subpopulations for basal, cycling, differentiating, or TSK tumor keratinocyte subpopulations (Table S2) after pre-processing data in a similar manner as patient data. During pre-processing, a small cluster of SCC-13 xenograft tumor cells expressing eccrine-associated genes could not be ruled out as possible murine cell contamination, thus were removed from further analysis. Unbiased clustering was performed in Seurat to generate four clusters from SCC-13 and CAL27 SCC cell line datasets (at resolution 0.15). Differential expression was then performed across these clusters and an overlap correlation matrix was generated from a binarized matrix of all DEGs with logFC > 0.25 and adjusted p value < 0.05 using the FindAllMarkers function in Seurat. For murine cells, datasets from SCC-13, CAL27, and A-431 tumors were merged and unbiased clustering was performed to generate DEG lists of markers, which were used to annotate cell types based on literature as described above for patient data cell annotation. These DEGs were converted to human genes and used to generate an overlap correlation matrix with analogous patient TME cell type DEGs (Figure 7E). Select shared DEGs from both patient and murine data are shown in Figures S7A and S7B. Because too few A-431 SCC cells were recovered from sequencing to perform similar analysis as the other two cell lines, we omitted murine cells derived from A-431 tumors in plots of xenograft TME data.

#### Spatial Transcriptomics Data Processing

The gene-spot matrices generated after ST data processing from ST and Visium samples were analyzed with the Seurat package (versions 3.0.0/3.1.3) in R in addition to custom scripts. For each patient data, spots were filtered for minimum detected gene count of 200 genes while genes with fewer than 10 read counts or expressed in fewer than 2 spots were removed. Normalization across spots was performed with the SCTransform function with regression of replicate and number of genes per spot. Dimensionality reduction and clustering was performed with independent component analysis (ICA) at resolution 0.8 with the first 20 ICs. For merged data shown in Figures S3B and S3C, all replicate samples across the four patients were merged with the “merge” Seurat function and re-normalized with SCTransform (regressing replicate and number of genes per spot) prior to ICA and UMAP on the first 20 ICs.

The spatial cluster gene signature overlap correlation matrix was generated by first taking all genes differentially expressed (average logFC > 0.25 and adjusted p value < 0.05 by Wilcoxon Rank Sum test; Table S4) across all ST clusters for each patient and generating a binarized matrix of genes and ST clusters (whether a gene belonged to the cluster or not). Pearson correlation was calculated across this matrix and hierarchical clustering of ST clusters was performed with the heatmap.2 function in the *gplots* package in R. Signature scoring derived from scRNA-seq or ST signatures was performed with the AddModuleScore function with default parameters in Seurat.

Nearest neighbor analysis was performed by counting the cluster identities of the four nearest neighbors on replicate sections for each ST section in the same patient. A null distribution of neighboring spots was determined by shuffling the cluster identities and counting nearest neighbor identities across randomized data for a total of 1,000 permutations while preserving number of spots and cluster identities per tissue section. The *P value* was the number of randomized permutations exceeding the observed data.

Spatial feature expression plots were generated with the SpatialFeaturePlot function in Seurat (version 3.1.3) and the STUtility R package (version 1.0.0).

#### Spatial Correlation Analysis

We reasoned that genes expressed in adjacent spots in ST were potentially meaningful and that a simple correlation of genes across spots could overlook this adjacency structure within the data. Thus, we calculated average normalized gene expression across a “sliding window” of spot groups consisting of a central spot surrounding by its N nearest neighbors, where N = 4 in the original ST data and N = 6 in Visium samples (illustrated in Figures S3A and S3J) for each spot in the tissue, generating a matrix of genes by average spot group expression across all spots. This matrix can be correlated with any “anchoring” gene of interest (*FOXP3* in our case) by calculating pairwise Pearson correlations of the *FOXP3* expression vector across all spots and the gene average group expression vectors across spots. These values reflect if the expression of a gene in the area surrounding the anchoring gene is correlated with the expression of the anchoring gene and termed “spatial gene correlation” with *FOXP3* as shown in Figures S5F and S5G.

#### Whole Exome Sequencing: analysis

Raw reads were aligned to hg19 using BWA with default parameters and duplicate reads were removed, resulting in a mean sequencing depth of 158x for on-target regions. The resulting aligned reads were processed using GATK 4.1.0.0 and Mutect2 variant calling for tumor-normal matched pairs, mostly following parameters from the GATK 4 documentation (https://gatk.broadinstitute.org). Mutect2 was run using a panel of normals derived from our cohort and the HapMap 3.3 allele frequencies as a germline resource. The resulting VCF files were filtered using FilterMutectCalls and FilterByOrientationBias and finally annotated using Funcotator, all within GATK tools.

#### Multiplexed Ion Beam Imaging Data Processing

##### Spectra calibration and file conversion

Mass-spectrometry pixel data was extracted into a multi-dimensional TIFF as previously described (Keren et al., 2018), using custom MATLAB (Mathworks, Inc., Natick, MA) scripts publicly available (https://github.com/lkeren/MIBIAnalysis). Time of flight (TOF) data was calibrated using sodium (Na, mass 22.99) and gold (Au, mass 196.97) peaks.

##### Background

Background subtraction of noise using bare regions of the slide with no tissues (high in Au counts) was performed as previously described (Keren et al., 2018).

##### Noise Removal

To filter noise by signal density a k-nearest-neighbor approach was used (Keren et al., 2018). In short, for each count, the average distance to the 25 nearest positive counts was counted. Pixels with counts larger than one were treated as several counts with distance 0 from each other. Removal thresholds were determined as the crossing points in the bimodal distributions and low-confidence counts were removed.

##### MIBI Image Visualization

MIBI images were converted to the MIBITiff format using custom scripts and uploaded onto the MIBItracker (V1.1.6.3, Ionpath Inc) for viewing. These images will be publicly available upon publication.

##### Segmentation

Nuclear segmentation was performed with DeepCell (Van Valen et al., 2016) (https://github.com/vanvalenlab/deepcell-tf) as previously described (Keren et al., 2018), using in-house training and validation MIBI imaging datasets from a variety of human clinical tissue types to ensure reproducibility.

##### MIBI Image Analysis

Feature counts per cell from the nuclear segmentation were extracted and normalized by cell size, before an arcsinh transformation with cofactor 1. A number of factors can potentially affect the staining and ion extraction efficiency on the MIBI-TOF, such as tissue sample fixation and preparation conditions, the Z-height of the samples, and local antibody concentrations. To adjust for these variations, we assumed that the median counts for the nucleus marker, dsDNA, should be fairly consistent across FOVs and patient tissue samples. All marker counts were normalized by the median dsDNA signal per FOV, with a cutoff of dsDNA > 0.1 to remove cells with minimal staining of dsDNA.

Marker expression was then scaled between the 10^th^ to 90^th^ percentiles, and then normalized on a 0 to 1 scale. Cell types were identified first using FlowSOM (Van Gassen et al., 2015) followed by Marker Enrichment Modeling (MEM) (Diggins et al., 2017), before visualizing using Uniform Manifold Approximation and Projection (UMAP) (McInnes et al., 2018).

For Figure 5B, the subset of markers consistent between MIBI and scRNA-seq for expression across cell types was presented (scRNA-seq data in Figure S5B). Hierarchical clustering in Figures 5C and 5D were performed using the R function heatmap.2 from the *gplots* package. To assess co-localization of pairs of non-tumor cell types, each FOV was analyzed to calculate the mean distance between each cell of cell type A and all cells of cell type B. For example, in Figure 5F, for each CD8 T cell, the mean distance to Tregs was calculated. To determine the significance of the distribution, the distance was calculated between each cell of cell type A and a random selection of non-tumor (and not A or B type) cells. This random selection was repeated 1,000 times, and a false discovery rate of observing a median distribution as extreme as the real observed distribution was calculated.

The identification of non-tumor cell location categories as tumor-infiltrated, tumor-stroma border (leading edge), or stromal was based on determining the identity of the 30 nearest neighbors to each non-tumor cell. The proportion of nearest neighbors flagged as tumor cells determined which category each cell fell: a cell having zero tumor cell neighbors flagged it as “stromal,” having between 5 and 13 tumor cell neighbors flagged it as “leading edge,” and having greater than 19 tumor cell neighbors flagged it as “infiltrated.”

#### Ligand-Receptor Interaction Analysis

Ligand-receptor interactions were inferred using a similar approach as previously described (Vento-Tormo et al., 2018). We first calculated average expression of ligand and receptor pairs across cell type pairs in normalized scRNA-seq data from an aggregate of the seven patient tumor samples containing TSK cells. We only considered genes with more than 10% of cells demonstrating expression within each cell type considered. We calculated a null distribution for average ligand-receptor by shuffling cell identities in the aggregated data and re-calculating ligand-receptor average pair expression across 1,000 permutations of randomized cell identities. The *P value* was the number of randomized pairs exceeding the observed data. For bar plots shown in Figures 6B and 6C, in addition to including only ligand-receptor pairs with p < 0.001, we further thresholded individual ligand or receptor expression with a cutoff of average expression > 0.2 (in log space). The 0.2 cutoff was determined by calculating the average log gene expression distribution for all genes across each cell type, and genes expressed at or above this cutoff corresponded with the top 12% or higher of expressed genes for each cell type.

For NicheNet (Browaeys et al., 2019) analysis, we derived TME cell type signatures by taking the top 100 differentially expressed genes in cells isolated from tumors or normal skin, including B cells, endothelial cells, fibroblasts, Langerhans cells, plasmacytoid DCs, CD1C DCs, CLEC9A DCs, T cells, NK cells, macrophages, and MDSCs. We input these signatures into NicheNet to derive a union set of predicted ligands modulating tumor-specific TME cell type signatures. For ligands predicting TSK modulation, we input the top 100 TSK-differentially expressed genes (Table S3). The top 15% of predicted ligands by regulatory potential that also demonstrated significance in our scRNA-seq ligand-receptor interaction analysis (described above) in each case are shown in Figures 6D and 6F. For differential expression testing of ligands and receptors in heatmaps from 6D and 6F, we used the FindAllMarkers function in Seurat to generate average logFC values per cell type compared to other cell types from the scRNA-seq data.

For ligand-receptor spatial transcriptomic proximity analysis, the average value of all ligand-receptor pairs across the leading edge from the eight sections from patients 2, 4, and 10 were calculated first by averaging the ligand and receptor expression among each leading edge spot and its 4-6 nearest neighbors (depending on ST technology), and then taking the average values of all of these groups of five or seven spots across the leading edge. This calculation for each ligand-receptor pair was then performed on 1,000 randomized permutations of spot identities while preserving total number of spots per replicate section to generate a null distribution per patient. *P value* was calculated by number of randomized permutation calculations that exceeded the true average.

#### CRISPR Guide Library Design

Subpopulation-enriched and SCENIC-nominated genes were selected based on analysis from patient scRNA-seq data (Figure S2F; Table S2). Merging patient and xenograft scRNA-seq helped select highly expressed genes across both datasets and exclude lowly expressed xenograft genes. Genes were determined to be subpopulation-enriched in patient scRNA-seq data if either one of two criteria were met: 1) recommended Seurat thresholds of average logFC > 0.25 and adjusted p value < 0.05 (Wilcoxon Rank Sum test) when compared to other subpopulations, or 2) average expression Z-score difference > 0.25 over any other subpopulation and adjusted p value < 0.05 (Wilcoxon Rank Sum test) when compared to other subpopulations. Genes not meeting these criteria were considered broadly expressed. Library guides were designed with Graphical User Interface for DNA Editing Screens (GUIDES) (guides.sanjanalab.org). The final library contained 334 genes at a coverage of eight guides per gene as well as 136 non-targeting control guides (Table S6).

#### CRISPR Screen Sequencing Analysis

Sequencing reads were first clipped using fastx clipper (from the FASTX-Toolkit package) for the sequence TCTTGTGGAAAGGACGAAACACCG. PCR duplicates were collapsed using the nine degenerate nucleotides added in PCR 1. Reads were then trimmed to the spacer sequence using fastx trimmer and mapped to the guide library using bowtie2. A read counts table was generated and used for downstream analysis.

The resulting matrix of read counts for each sgRNA (rows) and each sample (column) was then depth normalized so that each sample summed to ten million reads. These counts were log2-normalized, and the values for the time point 0 for each sgRNA were subtracted from time point 1 (three weeks of culture for *in vitro* and 8-10 weeks of xenograft growth for *in vivo*). Then for each sample, the median value of non-targeting sgRNAs was subtracted from all gene-targeting sgRNAs. These values are presented as “log_2_FC” in Figure 7 and Figure S7. We used the STARS algorithm to determine the false discovery rate for each gene (Doench et al., 2016). The log_2_FC tables were averaged across samples for each sgRNA and the resulting table was used as input to STARS, which was run with default parameters.

The significance of the difference of log_2_FC observed in *in vitro* and *in vivo* screens was determined using a permutation false discovery test (Figure S7D). On a gene by gene basis, the log_2_FC values for each *in vivo* tumor and *in vitro* biological replicate were permuted one thousand times. Then the number of instances of the permuted data exhibiting a more extreme difference between *in vivo* and *in vitro* was calculated, and this proportion was reported as the FDR.

#### Co-Essentiality Analysis

Co-essential genes were determined from Wainberg et al., 2019, which scored co-essentiality of genes across genome-wide CRISPR screens in 485 cancer cell lines (Meyers et al., 2017) using a generalized least-squares regression. To build our network, we included all co-essential gene pairs at their previously determined FDR < 0.10 that were non-syntenic. We seeded the network using any hit that was depleted in at least one of our screens at FDR < 0.10 as determined by the STARS algorithm. We included any other gene in the network that was annotated as co-essential with at least two of the seed genes. For network visualization, genes were represented by nodes, while edges represent a co-essential relationship at FDR < 0.10.

#### Analysis of TCGA Copy Number Variation and Expression

To determine the average proportion of samples of each tumor type exhibiting copy number variation of every gene, we first downloaded the Gistic2 gene-level thresholded copy number tables from the UCSC Xena database (https://xenabrowser.net/datapages/). We analyzed a collection of 31 solid tumors including ACC, BLCA, BRCA, CESC, CHOL, COAD, ESCA, GBM, HNSC, KICH, KIRC, KIRP, LGG, LIHC, LUAD, LUSC, MESO, OV. PAAD, PCPG, PRAD, READ, SARC, SKCM, STAD, TGCT, THCA, THYM, UCEC, UCS, UVM. For each tumor type, we calculated the proportion of tumors for each gene with a score less than or greater than 0 indicating copy number loss or gain, respectively. We then averaged these proportions across all tumor types to generate the values plotted in Figure S7G.

We analyzed correlations between inferred TSK abundance and individual TME cell type abundances first by identifying a set of genes for each cell type using the average expression of genes for that cell type in our scRNA data. Genes were considered specifically expressed if they exhibited an expression value greater than 0.8 (Seurat normalized values) in the cell type of interest and less than 0.2 in all other cell types (Figure S6D). These gene sets were then used to calculate a cell type module score, which was computed finding a set of 100 background genes in the same expression bin (the range of expression of all genes divided by 20) for each gene in the signature, and subtracting mean expression of this background gene set from the mean expression of the cell type gene set in each TCGA bulk tumor. We then calculated the Pearson correlation between the module score of a curated specific (Seurat p < 1e-10) TSK gene set (*MMP10*, *PTHLH*, *LAMC2*, *SLITRK6*) and the module scores of each TME cell type in each tumor type to produce the values presented in Figure S6E.

We interrogated coupled CNV and expression data to identify genes that exhibited genetic evidence of altered CAF abundance by first identifying tumor samples with copy number loss (Gistic2 thresholded value less zero, as in the CNV analysis above) versus samples with stable copy number status for each gene (Gistic2 value of zero). We also calculated the inferred CAF abundance by computing the module score for the CAF signature for each tumor, as described above. For each gene, we then subtracted the average CAF module score of CNV-loss tumors from CNV-stable tumors to determine the effect of CNV loss on CAF abundance. We also calculated the correlation between the expression of the same gene and the CAF module score, and these values (for each gene across all 31 tumor types) are presented in the plot in Figure 6H. To identify genes with statistically significant values of CNV-loss scores or correlations, we permuted the tumor sample IDs 100 times and calculated the range of CNV-loss scores and correlations in each permutation. We flagged genes as significant with values more extreme than 95% of the permuted values to establish a false discovery rate of 5%. Genes highlighted in the plot in Figure 6H are the subset of statistically significant genes that were predicted as TSK-specific ligands interacting with CAFs by the NicheNet analysis.

Kaplan-Meier plots shown in Figure S7H were generated using the Survival package in R. Tumors from TCGA types shown were scored with the average expression of the TSK gene set (*MMP10*, *PTHLH*, *LAMC2*, *SLITRK6*). Patients in the top and bottom 20% of scores were designated as TSK high and low, respectively. P value was calculated using a chi-square test.

#### Analysis of Gene Signature Associated with Response to Anti-PD-1 therapy

Gene expression and clinical data were downloaded from the supplemental data of Prat et al., 2017. Samples corresponding to head and neck SCC (HNSC) and squamous non-small cell lung cancer (LUSC) were analyzed for the mean expression of the two TSK-biased genes found in the select genes probed in this study: *ITGB1* and *PLAU*. Samples were stratified into high or low expression groups of this gene set (top and bottom 50% of samples, respectively, across all HNSC and LUSC samples). The progression-free survival after anti-PD-1 treatment was then analyzed using the Survival package in R to generate the Kaplan-Meier plot shown in Figure 6I and the p value was calculated using a chi-square test.

## Supplemental Information

Table S1. Patient Sequencing Data Summary, Related to Figure 1

Table S2. Keratinocyte Differentially Expressed Genes, Related to Figure 2

Table S3. TSK Signature Genes, Related to Figure 2

Table S4. ST Differentially Expressed Genes, Related to Figure 3

Table S5. MIBI Antibody Panel, Related to Figure 5

Table S6. CRISPR Library Guide List, Related to STAR Methods
