# Multimodal Analysis of Composition and Spatial Architecture in Human Squamous Cell Carcinoma

**DOI:** 10.1016/j.cell.2020.08.043

**Published:** 2020-09-17

**Authors:** Andrew L. Ji, Adam J. Rubin, Kim Thrane, Sizun Jiang, David L. Reynolds, Robin M. Meyers, Margaret G. Guo, Benson M. George, Annelie Mollbrink, Joseph Bergenstråhle, Ludvig Larsson, Yunhao Bai, Bokai Zhu, Aparna Bhaduri, Jordan M. Meyers, Xavier Rovira-Clavé, S. Tyler Hollmig, Sumaira Z. Aasi, Garry P. Nolan, Joakim Lundeberg, Paul A. Khavari

(Cell *182*, 497–514.e1–e22; July 23, 2020)

As a result of human error, the spatial feature plot labeled *ITGB1* in Figure 6G was instead a duplicate of the *ITGA3* data just below it. Additionally, in Figure S4C, the order of some of the bar labels on the plot were swapped, in particular, the order of Treg, CD4+ Naïve, NK, and CD8+ Naive. Both figures have been corrected online. We are confident that these inadvertent panel duplication and labeling errors did not have any effect on our analyses or on any conclusions drawn from the paper, and we apologize for the errors.

**Figure 6G:**
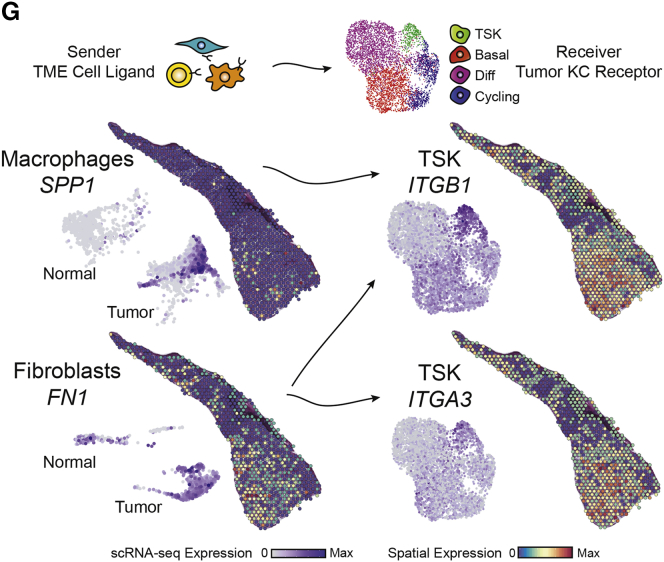
Cellular Crosstalk Landscape Associated with Leading Edge Niches (corrected)

**Figure 6G:**
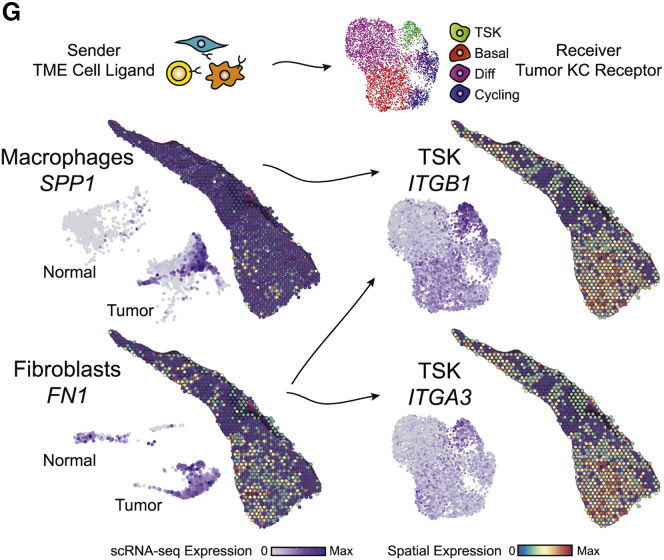
Cellular Crosstalk Landscape Associated with Leading Edge Niches (original)

**Figure S4C:**
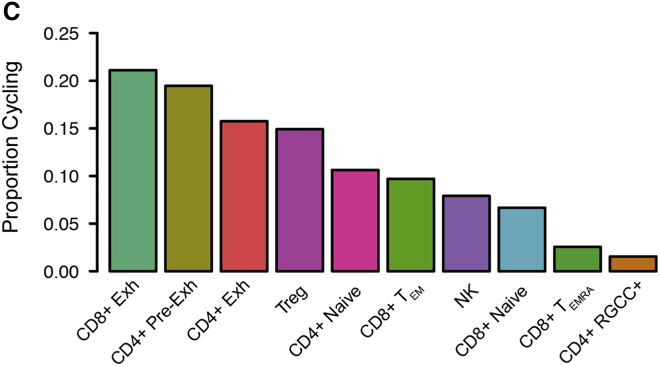
T Cell Subset Characterization and Spatial Positioning (corrected)

**Figure S4C:**
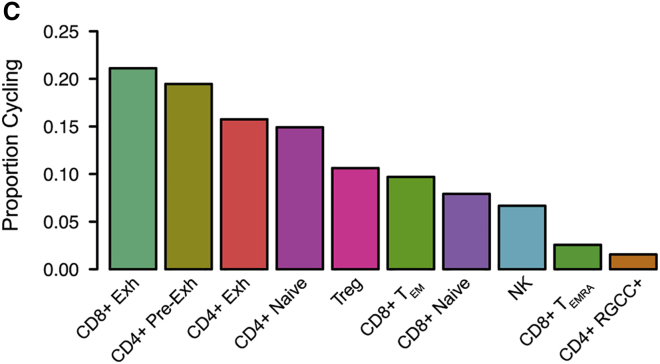
T Cell Subset Characterization and Spatial Positioning (original)

