# Supplementary material for: Multimodal Analysis of Composition and Spatial Architecture in Human Squamous Cell Carcinoma
Source: Cell. 2020 Jul 23;182(2):497–514.e22. doi: 10.1016/j.cell.2020.05.039 (PMC7391009; doi:10.1016/j.cell.2020.05.039)
Supplement: Table S5. MIBI Antibody Panel, Related to Figure 5 [file mmc5.docx]

Supplementary Table 5: MIBI Antibody Panel

| **Antibody** | **Clone** | **Source** | **Identifier** |
| --- | --- | --- | --- |
| dsDNA | 35I9 DNA | Abcam | ab27156 |
| Vimentin | D21H3 | Cell Signaling Technology | Custom Purified |
| Histone H3 | D1H2 | Cell Signaling Technology | Custom Purified |
| CD45RO | UCHL1 | Biolegend | 304202 |
| VISTA | D1L2G | Cell Signaling Technology | Custom Purified |
| MPO | AF3667 | Novus Bio | AF3667 |
| Lag3 | 17B4 | Lifespan Biosciences | LS-C18692 |
| CD4 | EPR6855 | Abcam | ab181724 |
| CD14 | EPR3653 | Abcam | ab214438 |
| CD56 | MRQ-42 | Cell Marque | Custom Purified |
| FoxP3 | 236A/E7 | Abcam | ab96048 |
| CD117 c-kit | C117/370 + KIT/982 + KIT/983 | Novus | NBP2-47845-0.1mg |
| Granzyme B | EPR20129-127 | Abcam | ab219803 |
| E-Cadherin | EP700Y | Abcam | ab201499 |
| Ki-67 | 8D5 | Cell Signaling Technology | Custom Purified |
| PD-1 | MRQ-22 | Cell Marque | Custom Purified |
| CD161 | ab197979 | Abcam | ab197979 |
| CD138 | EPR6454 | Abcam | ab212211 |
| CD163 | EDHu-1 | Novus | NB110-40686 |
| CD68 | D4B9C | Cell Signaling Technology | Custom Purified |
| Syndecan 4 | ab24511 | Abcam | ab24511 |
| CD3 | MRQ-39 | Cell Marque | Custom Purified |
| CD45-RA | HI100 | BD Biosciences | 555486 |
| CD42b | MM2/174 | Novus | NBP1-28457 |
| ICOS | D1K2T | Cell Signaling Technology | Custom Purified |
| CD21 | BU32 | Biolegend | 354902 |
| CD20 | rIGEL/773 | Novus | NBP2-53190-100ug |
| CD8 | C8/144B | Cell Marque | Custom Purified |
| HLA-DR | EPR3692 | Abcam | ab215985 |
| IL10 | 4A7-25-17 | Abcam | ab134742 |
| CD169 (Sialoadhesin) | SP213 | Abcam | ab245735 |
| CD34 | QBEnd/10 + HPCA1/763 | Novus | NBP2-47909 |
| Pan-Keratin | AE1/AE3 | Abcam | ab80826 |
| CD11b | EPR1344 | Abcam | ab216445 |
| CD38 | EPR4106 | Abcam | ab176886 |
| CD123 | 7G3 | BD Biosciences | 554529 |
| CD25 | 4C9 | Cell Marque | Custom Purified |
| CD45 | D9M8I | Cell Signaling Technology | Custom Purified |
